# Congenital and Breed-Associated Vertebral Anomalies in Dogs and Cats: Developmental Anatomy, Genetic Determinants, and Biological Significance

**DOI:** 10.3390/life16091544

**Published:** 2026-09-16

**Authors:** Costică Toader Covașă, Andrei Cristian Grădinaru

**Affiliations:** “Ion Ionescu de la Brad” Iasi University of Life Sciences, 3 Sadoveanu Alley, 700490 Iasi, Romania

**Keywords:** vertebral malformations, hemivertebra, axial development, brachycephalic dogs, screw tail, transitional vertebra, genotype–phenotype relationship, *DVL2*, *TBXT*

## Abstract

Congenital and breed-associated vertebral anomalies in dogs and cats encompass a heterogeneous group of abnormalities affecting vertebral formation, segmentation, regional identity, and number. Their interpretation requires integration of postnatal anatomy with developmental biology and genetic evidence, because similar morphological phenotypes may arise through different mechanisms and breed association does not necessarily imply a shared genetic cause. This structured narrative review synthesizes current knowledge on developmental anatomy, regional vertebral morphology, congenital and transitional anomalies, breed-associated phenotypes, and established genotype–phenotype relationships in dogs and cats. Particular attention is given to the distinction between morphological description and demonstrated developmental or molecular causation. Evidence involving *DVL2* in screw-tailed brachycephalic dogs and *TBXT* in naturally short-tailed dogs and Manx cats illustrates both the value and the limitations of single-gene explanations, including variable expressivity, allelic heterogeneity, and population specificity. Precise anatomical phenotyping emerges as a prerequisite for meaningful genetic analysis and breeding-oriented interpretation. Future progress will depend on standardized phenotype definitions, better-characterized populations, species-specific genetic studies, and functional validation of candidate mechanisms. Integrating developmental anatomy and genetics provides a rigorous framework for understanding the biological significance of congenital vertebral diversity in dogs and cats.

## 1. Introduction

The vertebral column constitutes the structural and functional axis of the vertebrate body, extending from the craniocervical junction to the terminal caudal region. It sustains posture and locomotion, protects the spinal cord and its coverings, and provides attachment sites for the muscles and ligaments responsible for axial stability and movement [1,2]. In dogs and cats, it is also a particularly informative developmental system in which regional specialization, congenital variation, axial patterning disorders, and breed-associated malformations converge [1,2,3].

The vertebral column is not a uniform osseous structure, but a segmented and regionally differentiated one. Its cervical, thoracic, lumbar, sacral, and caudal regions differ in morphology, mobility, and biomechanical function. The cervical spine supports the head and allows a wide range of motion. The thoracic spine articulates with the ribs and contributes to thoracic stability. The lumbar region balances flexibility with force transmission, whereas the fused sacrum connects the axial skeleton to the pelvic girdle. The caudal vertebrae form the most variable terminal segment of the column [1,2]. These regional distinctions are directly relevant to pathology because the anatomical context in which a congenital anomaly develops strongly shapes its morphology and functional consequences [1,2,3].

Normal regional anatomy offers more than a descriptive baseline. It provides the framework needed to understand how anomalies arising at specific axial levels may alter vertebral alignment, vertebral canal geometry, rib articulation, or force transmission [1,2,3]. A vertebral body defect in the thoracic region may affect spinal curvature and thoracic symmetry, whereas a comparable lesion at the lumbosacral junction may predominantly influence segmental stability or cauda equina function [3]. Likewise, abnormalities of the atlas, axis, or occipitocervical junction may have disproportionate consequences because of the specialized anatomy of the craniocervical region [3].

Interpretation of congenital vertebral anomalies also depends on vertebral development. The axial skeleton arises from the paraxial mesoderm through a coordinated sequence that includes somitogenesis, sclerotome differentiation, vertebral resegmentation, chondrogenesis, ossification, and specification of regional identity [4,5,6]. During this process, sclerotomal cells migrate around the notochord and neural tube and contribute to the vertebral bodies, neural arches, ribs, and components of the intervertebral discs. Resegmentation subsequently reorganizes adjacent sclerotomal domains to generate definitive vertebral elements and intervertebral boundaries [4,5,6].

Regional vertebral identity is established through conserved molecular mechanisms, particularly *HOX*-mediated patterning along the craniocaudal axis [7,8]. Disturbances occurring at different developmental stages may therefore give rise to distinct categories of vertebral pathology. Abnormal segmentation may produce numerical variation or fusion defects. Impaired vertebral body formation may lead to wedge-shaped vertebrae, ventral or lateral hypoplasia, and butterfly vertebrae. Defects of neural arch development may result in spina bifida or other forms of spinal dysraphism, whereas altered regional identity may manifest as cervical ribs or transitional vertebrae. Disruption of posterior axial development may produce sacrocaudal and caudal malformations, including shortened- or absent-tail phenotypes [3,6,7,8].

Congenital vertebral anomalies range from incidental anatomical variants to clinically significant malformations. Their relevance cannot be inferred from morphology alone because similar lesions may have different consequences depending on anatomical location, morphological severity, associated spinal deformity, and involvement of the spinal cord or cauda equina [3,9,10,11]. Radiography, computed tomography, and magnetic resonance imaging contribute to lesion recognition and characterization, but imaging findings must be interpreted within their anatomical and developmental context [9,10,11].

The domestic dog (*Canis lupus familiaris*) is particularly valuable for the study of breed-associated vertebral pathology because of its marked morphological diversity and highly structured breed populations [12,13,14]. Breeding practices and geographic isolation can contribute to differentiation among breed lineages and to reduced genetic diversity within individual populations [13,14], while breed-associated morphological diversity reflects the effects of strong selection on a comparatively restricted set of genetic loci [12]. Congenital axial abnormalities are especially common in brachycephalic screw-tailed breeds, although their distribution and morphological expression vary among breeds and individuals [9,10,11,15]. The relationship between breed-associated morphology and vertebral phenotype is exemplified by the frameshift variant identified in the *DVL2* gene in Bulldogs and related screw-tailed breeds. *DVL2* encodes the Dishevelled 2 protein, a component of WNT signaling, and the associated variant contributes to a Robinow-like phenotype involving caudal vertebral malformations and other skeletal characteristics [16,17]. The available evidence instead supports this variant as a breed-associated contributor to a specific axial phenotype rather than as a universal cause of hemivertebrae or other congenital vertebral malformations in dogs [16,17].

In cats (*Felis catus*), the most clearly established developmental-genetic association relevant to the caudal axis involves *TBXT*, historically designated *T* and encoding the transcription factor Brachyury. Mutant *TBXT* alleles have been associated with Manx short-tail phenotypes [18]. Phenotypic expression ranges from moderate tail shortening to complete taillessness and may include sacrocaudal, neural, and soft-tissue abnormalities [18,19]. In dogs, *TBXT* variants are associated with natural bobtail phenotypes in several breeds, although not all naturally short-tailed breeds share the same genetic basis [20,21].

For many canine and feline vertebral anomalies, however, the underlying genetic architecture remains incompletely understood [3,11]. Genes involved in somitogenesis, segmentation-clock regulation, sclerotome differentiation, chondrogenesis, ossification, and *HOX*-mediated regional identity provide biologically plausible candidate mechanisms [4,5,6,7,8]. Evidence derived from humans or experimental vertebrate models should therefore be distinguished from direct proof of causation in dogs or cats. Species-specific genetic associations, breed predisposition, familial aggregation, comparative developmental evidence, and mechanistic hypotheses require separate interpretation.

Congenital and breed-associated vertebral anomalies raise breeding and animal-welfare considerations, particularly when externally selected conformational traits coexist with internal axial malformations [10,11,15,16,17]. More broadly, canine breed formation and artificial selection have generated marked conformational differentiation [12,13,14], while specific genetic variants contribute to screw-tail and natural bobtail phenotypes [16,17,20,21]. Extreme brachycephalic conformation provides a separate example of how strongly selected morphology may be associated with adverse health consequences [22]. These considerations reinforce the need to distinguish an externally visible breed characteristic from an incidental anatomical variant and from a clinically significant malformation.

Accordingly, this narrative review integrates the regional anatomy of the canine and feline vertebral column with the developmental pathology, morphological classification, genetic evidence, and breeding implications of congenital and breed-associated vertebral anomalies. Diagnostic imaging and clinical consequences are considered when they contribute directly to morphological interpretation and pathological significance. Particular attention is given to distinguishing incidental anatomical variants from developmental malformations and validated genetic associations from candidate or comparative mechanisms.

## 2. Review Methodology

### 2.1. Review Design and Scope

This structured narrative review examines congenital and breed-associated vertebral anomalies in domestic dogs (*Canis lupus familiaris*) and cats (*Felis catus*) by synthesizing evidence from vertebral anatomy, developmental biology, pathology, and genetics. The methodological structure and reporting of the review were informed by the Scale for the Assessment of Narrative Review Articles (SANRA) [23].

Normal vertebral anatomy served as the reference framework for recognizing abnormal morphology and interpreting the regional consequences of developmental defects. Developmental evidence was considered in relation to somitogenesis, sclerotome differentiation, vertebral resegmentation, chondrogenesis, ossification, and craniocaudal patterning. Genetic evidence was evaluated with emphasis on breed predisposition, familial aggregation, molecular associations, and genotype–phenotype relationships. The term “vertebral anomaly” was used broadly to encompass numerical variation, altered regional identity, segmentation and fusion defects, vertebral body malformations, neural arch abnormalities, and disorders affecting the lumbosacral, sacrocaudal, and caudal regions. More specific terms, including malformation, aplasia, hypoplasia, dysplasia, fusion, dysraphism, and transitional vertebra, were used when supported by the morphological or pathological findings reported in the original publication.

### 2.2. Literature Identification and Selection

Relevant literature was identified through structured, topic-specific searches of PubMed/MEDLINE, Scopus, Web of Science Core Collection, and Google Scholar. No lower publication-date limit was applied; records available from database inception through 9 August 2026 were considered. Search syntax was adapted to the requirements of each database and to the anatomical region or phenotype under consideration. Representative search combinations included: (dog OR canine OR “*Canis lupus familiaris*”) AND (“vertebral anomaly” OR “vertebral malformation” OR hemivertebra OR “block vertebra” OR “transitional vertebra” OR “spinal dysraphism” OR “spina bifida”); (cat OR feline OR “*Felis catus*”) AND (“vertebral anomaly” OR “vertebral malformation” OR “transitional vertebra” OR “sacrocaudal dysgenesis” OR “spinal dysraphism”); and (dog OR cat OR canine OR feline) AND (DVL2 OR TBXT OR NKX2-8 OR genetic* OR inherit* OR “genotype–phenotype”) AND (vertebr* OR tail OR “axial skeleton”). Additional developmental searches combined terms such as somitogenesis, sclerotome, resegmentation, segmentation clock, HOX, chondrogenesis, and vertebral development. Google Scholar was used primarily for supplementary retrieval and citation tracing.

Publications were eligible when they provided information directly relevant to at least one of the predefined review domains: (i) congenital or breed-associated vertebral morphology in dogs or cats; (ii) anatomical, radiographic, computed tomographic, magnetic-resonance, pathological, or developmental characterization of a vertebral phenotype; (iii) breed distribution, familial aggregation, heritability, inheritance, or molecular genotype–phenotype relationships; or (iv) comparative developmental or molecular evidence needed to interpret conserved mechanisms of vertebral formation. Peer-reviewed canine and feline studies were prioritized. Authoritative veterinary anatomy and developmental biology textbooks were retained for foundational anatomical information, and human or experimental vertebrate studies were included selectively when they addressed conserved developmental mechanisms relevant to the phenotypes reviewed.

Records were excluded when they addressed exclusively acquired, traumatic, degenerative, infectious, or neoplastic spinal disease without relevance to congenital vertebral morphology; lacked sufficient anatomical, phenotypic, developmental, or genetic information for the purposes of the review; duplicated a publication already represented by a more complete report; or could not be adequately verified from the available bibliographic record or full text. Non-peer-reviewed material was not used as primary scientific evidence, apart from the authoritative textbooks used for baseline anatomy.

Potentially relevant records were screened first by title and abstract and subsequently by full-text assessment. Reference lists of relevant primary studies and recent reviews were cross-checked to identify additional eligible publications. Final inclusion was based on relevance to the predefined anatomical, developmental, pathological, or genetic domains and on the ability of a source to support a specific statement or comparison in the review. Both authors participated in literature identification, source verification, and final selection. Because this structured narrative review used iterative, topic-specific searches and citation tracing rather than a single prospectively registered systematic search, a single aggregate number of records initially retrieved across all search iterations was not prospectively recorded. The substantial overlap among database queries, supplementary searches, and citation tracing also precludes reliable retrospective reconstruction of a unique initial record count without double-counting. The final evidence base comprised 85 cited sources, including 83 journal articles and two authoritative veterinary anatomy textbooks. The final literature search and web-based reference verification were completed on 9 August 2026.

### 2.3. Evidence Appraisal and Terminology

Evidence was interpreted according to its source, study design, and directness. Species-specific molecular findings were considered strongest when a genetic variant was supported by association or segregation data and, where available, by functional evidence or reproducible genotype–phenotype relationships. Recurrent occurrence within a breed or family without identification of a molecular determinant was interpreted as evidence of predisposition or possible inheritance rather than proof of a defined mode of inheritance.

Anatomical and pathological findings were used to define lesion morphology, regional distribution, and structural significance but were not considered sufficient to demonstrate genetic causation. Similarly, developmental mechanisms identified in humans or experimental vertebrate models were used to support biological plausibility and were distinguished from relationships demonstrated directly in dogs or cats.

Reported frequencies were interpreted in relation to breed composition, population characteristics, selection criteria, and the definitions used for vertebral anomalies. Frequencies obtained from selected clinical, referral, or breed-specific populations were not regarded as equivalent to population-level prevalence.

Terminology was standardized according to morphological and developmental criteria. The term “hemivertebra” was not used indiscriminately for all vertebral body malformations when more precise descriptions, such as ventral hypoplasia, lateral hypoplasia, wedge-shaped vertebra, or butterfly vertebra, were available.

Causal terminology was used conservatively. Breed predisposition was distinguished from demonstrated heritability or a defined mode of inheritance, and comparative candidate mechanisms were distinguished from species-specific genetic associations. Terms such as “associated with”, “predisposed to”, “candidate mechanism”, and “causal variant” were used according to the strength and directness of the available evidence.

### 2.4. Use of Generative Artificial Intelligence

**Figure 1:**
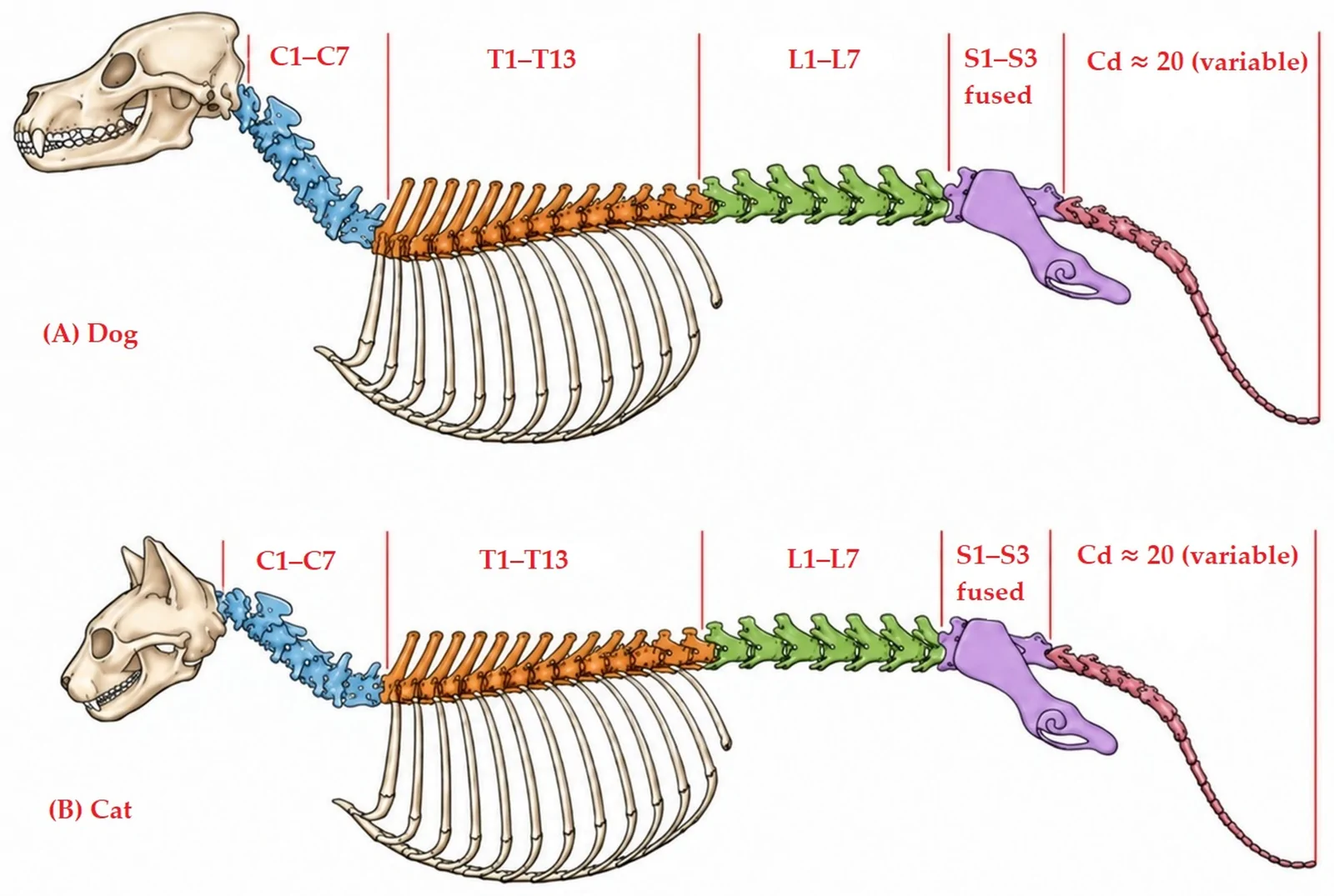
Typical regional organization of the vertebral column in dogs and cats. (**A**) Dog; (**B**) cat. Both species usually have seven cervical (C1–C7), thirteen thoracic (T1–T13), seven lumbar (L1–L7), and three fused sacral vertebrae (S1–S3); the caudal (Cd) series is numerically variable and generally comprises approximately 20 vertebrae. Transitional morphology may modify regional identity and apparent vertebral counts at axial boundaries [1,2,32,33]. The illustration is schematic and not to scale.

During manuscript preparation and revision, OpenAI ChatGPT (GPT-5.6 Sol) was used as an AI-assisted tool for English-language refinement, editorial review, and assistance in preparing the schematic illustration presented as Figure 1. The scientific content, literature selection, interpretation of the evidence, anatomical organization, vertebral regions, regional vertebral counts, figure labels, and explanatory content were determined and verified by the authors on the basis of the original sources cited in the manuscript. All AI-assisted textual and graphical output was critically reviewed, edited where necessary, and approved by the authors, who take full responsibility for the accuracy, integrity, and final content of the manuscript.

## 3. Developmental Anatomy and Genetic Basis of Vertebral Malformations

The vertebral column develops through a tightly coordinated sequence of anatomical and molecular events that transforms the paraxial mesoderm into a segmented and regionally differentiated axial skeleton. Somite formation is followed by sclerotome differentiation, vertebral resegmentation, chondrogenesis, ossification, and the establishment of regional vertebral identity [4,5,6,24]. Disturbances occurring at different stages of this sequence may produce distinct pathological phenotypes, including segmentation and fusion defects, abnormalities of vertebral body or neural arch formation, transitional vertebrae, and malformations of the sacrocaudal and caudal axis [3,4,5,6,24].

### 3.1. Somite Formation, Sclerotome Differentiation, and Vertebral Resegmentation

The metameric organization of the vertebral column originates from somites, which arise sequentially from the presomitic paraxial mesoderm along the craniocaudal axis. Each somite differentiates into a dermomyotome and a sclerotome. The dermomyotome contributes primarily to skeletal musculature and dermis, whereas the sclerotome gives rise to the vertebral bodies, neural arches, ribs, and part of the intervertebral discs [4,6,24].

Sclerotomal cells migrate medially around the notochord and dorsally around the developing neural tube. Their spatial distribution establishes the primordia of the vertebral body and neural arch. This developmental arrangement is relevant to pathological interpretation because defects affecting ventromedial sclerotomal development may predominantly involve the vertebral body, whereas disturbances of dorsal migration or differentiation may result in abnormalities of the neural arches and other posterior vertebral elements [4,6,24].

A central event in vertebral morphogenesis is resegmentation. In the classical model, the caudal portion of one sclerotome combines with the cranial portion of the adjacent sclerotome to form a definitive vertebra. This arrangement positions the intervertebral disc at the original intersomitic boundary, permits spinal nerves to emerge between adjacent vertebrae, and allows segmental muscles to span the intervertebral joints [4,5,24]. More recent developmental evidence indicates that vertebral patterning is more complex than the simple fusion of two equivalent sclerotomal halves. Vertebral bodies and neural arches form through resegmentation, while sclerotomal cells also undergo regionally variable shifts according to their position within the developing segment [5]. This refined model is particularly relevant to congenital pathology because an abnormal vertebra may show variable involvement of the centrum, neural arch, articular processes, or adjacent vertebral boundaries. Ward et al. [5] specifically demonstrated resegmentation of both vertebral bodies and neural arches and proposed the resegmentation-shift model on this basis.

Disruption of somite boundary formation or resegmentation may result in incomplete vertebral separation and congenital fusion between adjacent vertebral elements, morphologically expressed as block vertebrae, asymmetric fusion, or more complex segmentation defects. Abnormal development of the vertebral primordium may instead produce absent or hypoplastic vertebral components. The resulting morphology depends on the developmental stage, anatomical compartment, and axial level involved [3,4,5,6,24].

### 3.2. Molecular Control of Vertebral Formation

Somitogenesis is regulated by coordinated oscillatory gene expression within the presomitic mesoderm. This process is commonly described by the segmentation clock and wavefront model, in which cyclic molecular activity interacts with positional gradients to determine when and where new somite boundaries are formed [25,26]. Notch, WNT/β-catenin, and fibroblast growth factor signaling are major components of this regulatory network. Together, they influence somite periodicity, boundary formation, bilateral symmetry, and rostrocaudal polarity. Disruption of these mechanisms may alter the number, shape, or separation of embryonic segments and thereby predispose to vertebral segmentation defects [25,26].

After somite formation, sclerotomal differentiation depends on signals originating from the notochord, neural tube, surface ectoderm, and adjacent mesoderm. Sonic hedgehog signaling from the notochord and floor plate promotes sclerotomal identity and vertebral chondrogenesis, whereas bone morphogenetic protein and WNT pathways contribute to the spatial organization and differentiation of somitic cell populations [4,6,24].

These signaling pathways form an interacting developmental network rather than isolated determinants of individual vertebral anomalies, contributing to the regulation of somite patterning, vertebral primordium formation, and cartilage template development. Disturbance of this network may affect segmentation, cell migration, chondrogenic differentiation, or the spatial relationship between the developing vertebra and the neural tube [4,6,24,25,26]. The resulting pathological phenotype may therefore reflect more than one developmental mechanism. A malformed vertebral body may arise from abnormal segmentation, deficient sclerotomal contribution, disturbed chondrogenesis, or asymmetric formation of the cartilaginous centrum. Likewise, abnormalities traditionally grouped under the term “hemivertebra” may reflect different developmental routes rather than a single embryological entity [4,5,6,24].

### 3.3. Axial Identity and Regional Vertebral Patterning

Segmentation establishes the repeated organization of the vertebral column, but it does not determine whether an individual vertebra acquires cervical, thoracic, lumbar, sacral, or caudal characteristics. Regional identity is specified along the craniocaudal axis primarily through coordinated *HOX* gene expression [7,8,27]. *HOX*-related patterning assigns positional information to the developing sclerotomes. Altered expression boundaries may lead to homeotic transformation, in which a vertebra develops morphological characteristics normally associated with an adjacent region [7,8,27]. Transitional vertebrae can therefore be interpreted developmentally as alterations or incomplete transformations of regional identity rather than exclusively as numerical abnormalities.

The strong conservation of seven cervical vertebrae in most mammals illustrates the developmental constraints acting on axial patterning [28,29]. Changes at the cervicothoracic boundary may be expressed through partial thoracization of C7, formation of cervical ribs, or other cervicothoracic transitional morphologies. These changes may occur without alteration of the total presacral vertebral number because regional identity and vertebral number are related but distinct developmental properties [28,29].

Domestic dogs provide a relevant example of this relationship. Cervical ribs and C7 morphology consistent with homeotic transformation have been documented particularly frequently in Pugs and have been reported together with altered presacral vertebral counts and other transitional vertebral morphologies [30]. Although the underlying genetic mechanism has not been established directly, the phenotype is anatomically consistent with altered specification of the cervicothoracic boundary. Brocal et al. [30] found cervical ribs in 45 of 97 Pugs and demonstrated associations with thoracolumbar transitional morphology and altered presacral vertebral count, supporting a morphological interpretation of the rib-bearing C7 phenotype as a partial homeotic transformation toward thoracic identity [30].

Comparable principles apply at the thoracolumbar and lumbosacral junctions. A vertebra situated at a regional boundary may display mixed morphological characteristics, such as incomplete rib formation, altered transverse processes, or partial incorporation into the sacrum. Their interpretation depends on the regional anatomy and structural relationships of the affected junction rather than on vertebral number alone [1,2,7,8,27,30].

### 3.4. Species-Specific and Comparative Genetic Evidence

The genetic architecture of congenital vertebral malformations is heterogeneous. In humans, pathogenic variants affecting segmentation-clock genes and related developmental regulators have been associated with spondylocostal dysostosis, congenital scoliosis, vertebral fusion, and other vertebral segmentation defects [31]. Genes implicated in these developmental disorders include *DLL3*, *MESP2*, *LFNG*, *HES7*, *TBX6*, and *RIPPLY2* [31]. These genes identify biological pathways relevant to vertebral development, but their involvement in human disease does not demonstrate that homologous genes cause morphologically similar vertebral anomalies in dogs or cats. In veterinary species, these genes therefore represent comparative candidate determinants in the absence of species-specific genetic evidence. The diversity of implicated developmental genes further illustrates the considerable genetic heterogeneity of congenital vertebral malformations [31].

Among the best-characterized species-specific genetic associations in domestic carnivores are variants associated with distinct breed-associated axial phenotypes. In dogs, a frameshift variant in *DVL2* has been associated with the screw-tail phenotype and broader Robinow-like skeletal characteristics in Bulldogs, French Bulldogs, and Boston Terriers [16,17]. The DVL2 protein participates in WNT signaling, thereby linking a validated breed-associated variant with a developmental pathway involved in axial morphogenesis. The *DVL2* variant is associated with a specific constellation of breed-associated morphological traits rather than with vertebral body malformations in brachycephalic dogs as a uniform category. Vertebral anomalies observed in other breeds, as well as different types of vertebral malformation within the same breeds, may have distinct or incompletely resolved genetic and developmental bases [16,17]. The contribution of additional genetic factors remains unresolved.

A second major example involves *TBXT*. Mutant *TBXT* alleles have been associated with Manx short-tail phenotypes in cats [18]. The degree of caudal reduction varies, and the external tail phenotype may be accompanied by variable sacrocaudal and neural involvement [18,19]. In dogs, *TBXT* variants are associated with natural bobtail phenotypes in several breeds [20,21]. However, the same variant is not present in all naturally short-tailed breeds [21]. Morphologically similar caudal phenotypes may therefore have genetically distinct molecular bases.

The available evidence consequently supports a hierarchical interpretation. *DVL2*- and *TBXT*-associated phenotypes represent directly investigated species-specific genotype–phenotype relationships [16,17,18,19,20,21]. Recurrent occurrence of a vertebral phenotype within a breed or family without an identified causative variant supports breed predisposition or possible inheritance but does not establish a defined mode of inheritance. By contrast, genes identified primarily through human disease or experimental models provide comparative biological plausibility and represent candidate determinants in dogs and cats in the absence of species-specific evidence [31].

### 3.5. Developmental–Pathological Interpretation of Vertebral Anomalies

The anatomical appearance of a vertebral anomaly represents the endpoint of a developmental disturbance and does not, by itself, define the underlying mechanism. Nevertheless, developmental anatomy provides a useful framework for relating morphology to its developmentally plausible origin [3,4,5,6,24].

Defects affecting somite formation or boundary specification are compatible with generalized or complex vertebral segmentation abnormalities. Disturbed resegmentation may contribute to incomplete vertebral separation and congenital fusion between adjacent vertebral elements. Defective formation of the vertebral body, or centrum, may produce hypoplastic, wedge-shaped, or asymmetrical vertebrae. Failure of normal development of the dorsal vertebral components may result in neural arch defects and spinal dysraphism [3,4,5,6,24]. These relationships provide developmental hypotheses rather than proof of the embryological mechanism responsible for an individual postnatal lesion.

Altered regional identity provides a developmental framework for interpreting cervical ribs and transitional vertebrae [7,8,27,30]. Disturbances of posterior axial development may be expressed as sacrocaudal dysgenesis and tailless or short-tail phenotypes in cats [18,19], whereas in dogs they include genetically associated natural bobtail and screw-tail phenotypes [16,17,20,21]. Specific genetic associations have been demonstrated for some of these conditions, whereas the molecular basis of others remains unresolved.

A developmental classification is therefore most informative when it combines three levels of interpretation: (i) the anatomical structure affected, such as the vertebral body, neural arch, intervertebral boundary, or regional junction; (ii) the developmentally plausible process involved, including segmentation, resegmentation, chondrogenesis, axial identity, or posterior axis formation; and (iii) the strength of the genetic evidence, which may range from a validated species-specific variant to breed predisposition or comparative biological plausibility. This framework avoids assigning a single developmental or genetic cause to all lesions with a similar external appearance. The principal developmental processes, associated vertebral phenotypes, and levels of genetic evidence discussed in this chapter are summarized in Table 1.

## 4. Regional Anatomy as a Framework for Developmental and Pathological Interpretation

The vertebral column of dogs and cats is organized into cervical, thoracic, lumbar, sacral, and caudal regions, each characterized by a distinct combination of morphology, mobility, and biomechanical function [1,2,32,33]. This regional differentiation provides the anatomical framework for interpreting congenital vertebral anomalies because the structural expression and pathological consequences of a developmental defect depend substantially on the axial level at which it occurs. The typical regional vertebral formula and the principal sources of numerical variation are summarized schematically in Figure 1.

### 4.1. Cervical Region

In dogs and cats, as in most mammals, the cervical region consists of seven vertebrae. The conservation of this numerical pattern reflects strong evolutionary and developmental constraints on mammalian cervical identity [28,29]. Alterations at the cervicothoracic boundary are therefore typically expressed not as major changes in cervical number, but as partial transformations of regional identity, including thoracization of C7, formation of cervical ribs, or other cervicothoracic transitional morphologies [28,29,30].

The atlas and axis are morphologically distinct from the remaining cervical vertebrae. The atlas lacks a conventional vertebral body and is formed predominantly by dorsal and ventral arches connected by lateral masses. Its expanded wings provide attachment surfaces and contain or border passages for neurovascular structures. The axis possesses a well-developed vertebral body, a prominent spinous process, and the dens, which projects cranially and forms the pivot of the atlantoaxial articulation [1,2,32,33]. This specialization permits mobility at the craniocervical junction while maintaining alignment among the skull, vertebral canal, and cranial cervical spinal cord. It also creates a region in which relatively localized developmental abnormalities may have marked structural consequences. Defects involving the atlas arches, formation of the dens, or the craniocervical articulations may alter the stability or geometry of this specialized junction [3].

The middle cervical vertebrae, C3–C6, conform more closely to the general vertebral pattern. They possess a vertebral body, vertebral arch, spinous and transverse processes, and paired articular processes. Their morphology balances cervical mobility with protection of the spinal cord and vertebral neurovascular structures. Morphological differences between dogs and cats occur mainly in vertebral proportions and process architecture, with feline vertebrae generally being more slender and differing in proportional details from canine vertebrae [1,2,32,33].

C7 marks the transition between the cervical and thoracic regions. Its transverse processes differ from those of the preceding cervical vertebrae, and its morphology reflects its position at a developmental boundary. Partial acquisition of thoracic characteristics may produce costal elements or transitional morphology without necessarily altering the total presacral vertebral number [28,29,30]. This boundary is particularly relevant in dogs because cervical ribs and homeotic transformation of C7 have been reported particularly frequently in Pugs [30]. These findings support the interpretation of cervicothoracic transitional morphology as a disturbance of regional identity rather than merely as numerical variation [7,8,27,30]. The phenotype is anatomically consistent with altered regional identity, while a direct genetic determinant has not been demonstrated [30].

From a developmental–pathological perspective, the cervical region contains two distinct zones of vulnerability. The atlas and axis are susceptible to anomalies affecting specialized vertebral components and craniocervical articulation [3], whereas C7 is susceptible to incomplete transformation of regional identity at the cervicothoracic boundary [7,8,27,30]. The intervening C3–C6 vertebrae retain the more typical cervical pattern between these two anatomically and developmentally specialized regions [1,2,32,33].

### 4.2. Thoracic Region

In dogs and cats, the thoracic region normally consists of thirteen vertebrae. Thoracic vertebrae are distinguished by their articulation with the ribs and their integration into the thoracic cage [1,2,32,33]. Typical thoracic vertebrae possess cranial and caudal costal foveae on the vertebral bodies for articulation with the heads of the ribs, together with transverse costal foveae for articulation with the rib tubercles. These morphological relationships differentiate the thoracic region from the adjacent cervical and lumbar segments [1,2,32,33].

The integration of the thoracic vertebrae with the ribs, costovertebral joints, intercostal structures, and sternum limits intervertebral mobility and provides relative axial stability. The thoracic region therefore functions as a combined vertebral and costal unit rather than as an isolated series of spinal elements. This structural arrangement is pathologically relevant because congenital abnormalities of vertebral formation may simultaneously alter rib orientation, thoracic symmetry, and vertebral column alignment [1,2,3,32,33].

The spinous processes are generally long and display a characteristic regional change in orientation. Cranial to the anticlinal vertebra, they are directed caudally, whereas those situated caudal to it become progressively more cranially inclined. The anticlinal vertebra, defined by the relatively vertical orientation of its spinous process, is usually T11 in dogs and cats, although variation in its radiographic identification has been documented in dogs [1,2,32,33,34].

T1 represents the cervicothoracic transition and possesses a prominent spinous process together with well-developed costal articulations. Toward the caudal thoracic region, the vertebrae progressively acquire morphological characteristics associated with the lumbar spine, including increasingly evident mammillary and accessory processes. The thoracolumbar junction therefore represents a gradual anatomical transition rather than an abrupt boundary [1,2,32,33].

The thoracic region is a frequent site of congenital vertebral body malformations in brachycephalic screw-tailed dog breeds [9,10,11,15]. Developmental defects may involve the entire vertebral body or predominantly its ventral, lateral, or central components, resulting in wedge-shaped vertebrae, ventral or lateral hypoplasia, butterfly vertebrae, and related asymmetrical configurations. Consequently, the traditional designation “hemivertebra” is less anatomically specific than descriptions based on the vertebral component affected, such as ventral or lateral hypoplasia, wedge-shaped vertebra, or butterfly vertebra [9,10,11].

Because thoracic vertebrae are structurally connected to the ribs, asymmetric vertebral development may be expressed not only as a localized vertebral deformity but also as altered spinal curvature, rib displacement, or thoracic asymmetry [3,9,10,11]. The pathological impact depends on the vertebral component affected, the direction and degree of asymmetry, the number and distribution of malformed vertebrae, and their relationship with adjacent normally formed segments [3,9,10,11].

Breed-associated clustering is particularly evident in French Bulldogs, English Bulldogs, Boston Terriers, and Pugs, although the prevalence, distribution, and morphology of thoracic anomalies vary among these breeds [9,10,11,15]. Although the *DVL2* variant contributes to the broader axial phenotype of several screw-tailed breeds, its association does not account for all thoracic vertebral body malformations [16,17]. Other vertebral phenotypes within these breeds may have distinct or incompletely resolved developmental and genetic bases [9,10,11,15,16,17].

From a developmental–pathological perspective, the thoracic region presents two principal areas of vulnerability. The vertebral body–rib complex is susceptible to asymmetrical formation defects that may modify both spinal and thoracic morphology [3,9,10,11]. The thoracolumbar junction, by contrast, is susceptible to transitional morphology compatible with incomplete specification of regional vertebral identity [7,8,27]. These relationships provide the anatomical framework for the subsequent classification of thoracic and thoracolumbar anomalies.

### 4.3. Lumbar Region

In dogs and cats, the lumbar region normally consists of seven vertebrae. Lumbar vertebrae are characterized by relatively large vertebral bodies, robust vertebral arches, prominent transverse processes, and well-developed articular, mammillary, and accessory processes [1,2,32,33]. These morphological features reflect the combined functional requirements of axial support, muscular attachment, controlled mobility, and force transmission between the thoracic region and the pelvis [35].

The lumbar vertebral bodies are relatively elongated and generally increase in size toward the caudal part of the series. Their broad end surfaces and intervening intervertebral discs contribute to load distribution, whereas the configuration of the articular processes permits substantial flexion and extension while limiting excessive axial rotation [1,2,32,33,35]. The accessory and mammillary processes provide additional attachment sites for the epaxial musculature and contribute to segmental stabilization [1,2,32,33].

Morphological differences between dogs and cats occur primarily in vertebral proportions and process architecture. Feline lumbar vertebrae generally exhibit relatively elongated bodies and longer, more gracile transverse processes, whereas canine morphology varies according to body size and conformation [1,2,32,33]. These differences do not alter the basic regional organization.

The lumbar region occupies an intermediate position between the relatively constrained thoracic spine and the rigid sacrum. Its cranial boundary is represented by the gradual thoracolumbar transition, whereas L7 forms the mobile component of the lumbosacral junction. This position makes the lumbar spine particularly relevant for interpreting variation in vertebral number, regional identity, and the structural relationships between the terminal lumbar vertebra and the sacrum [1,2,7,8,27,32,33].

At the thoracolumbar boundary, transitional morphology may be expressed as incomplete development of a rib, formation of an elongated transverse process, or the presence of mixed thoracic and lumbar characteristics. Such configurations are developmentally compatible with incomplete specification of regional identity rather than representing purely numerical variation [7,8,27]. Precise morphological classification incorporates both the configuration of the transitional vertebra and its relationships with the adjacent ribs and vertebral segments.

The lumbosacral junction represents a second region of developmental and structural vulnerability. Partial sacralization of L7, lumbarization of the first sacral segment, or asymmetric incorporation of a transitional vertebra into the sacrum may modify the normal boundary between the mobile lumbar column and the fused sacral region. Such configurations are generally classified as lumbosacral transitional vertebrae, although their morphology and degree of sacral incorporation may vary [1,2,3,7,8,27,36,37].

The pathological significance of lumbar and lumbosacral anomalies depends on more than their presence alone. Relevant structural features include vertebral symmetry, axial alignment, formation of the articular processes, relationships with the sacrum and pelvis, and the distribution of mechanical loading across adjacent segments [1,2,3,35,36,37]. Consequently, the presence of a transitional vertebra alone does not establish it as the primary cause of neurological or locomotor dysfunction [3,36,37].

From a developmental–pathological perspective, the lumbar region contains two principal transitional domains. The thoracolumbar junction is susceptible to transitional configurations compatible with altered thoracic–lumbar regional identity, whereas the lumbosacral junction may show incomplete lumbar–sacral transformation and asymmetric incorporation into the sacrum [7,8,27]. Between these boundaries, the lumbar vertebrae form a mobile, load-bearing unit whose morphology influences the structural consequences of congenital vertebral abnormalities [1,2,32,33,35].

### 4.4. Sacral Region

In adult dogs and cats, the sacrum is normally formed by the fusion of three sacral vertebrae, S1–S3. It is a relatively short and compact structure, with a broad cranial base, a narrower caudal apex, paired wings, dorsal and pelvic surfaces, a sacral canal, and dorsal and ventral sacral foramina. Its lateral auricular surfaces articulate with the ilia to form the sacroiliac joints, establishing the osseous connection between the vertebral column and the pelvic girdle [1,2,32,33].

Through the sacroiliac joints, the sacrum transmits forces between the pelvic limbs and the axial skeleton and contributes to pelvic stability [1,2,32,33,35]. Fusion of the sacral segments provides the rigidity required for this function and distinguishes the sacrum from the mobile lumbar and caudal regions [1,2,32,33].

Although the mature sacrum functions as a single osseous unit, its morphology reflects the development, regional specification, and subsequent fusion of three initially segmental vertebral primordia [4,5,6,24]. Alterations involving these developmental processes are compatible with incomplete fusion, asymmetrical sacral formation, abnormal incorporation of an adjacent vertebra, or altered structural relationships between the sacrum and the pelvis [7,8,27].

The cranial sacral boundary is defined by the lumbosacral junction between L7 and S1. Altered or incomplete specification of lumbar versus sacral identity provides a developmental framework for interpreting sacralization of L7, lumbarization of S1, and intermediate vertebral morphologies combining lumbar and sacral characteristics [7,8,27]. Such configurations are classified as lumbosacral transitional vertebrae. Their morphological expression ranges from enlargement or altered orientation of the transverse processes to unilateral or bilateral articulation or fusion with the sacrum or ilium [3,36,37].

Asymmetrical incorporation of a transitional segment may modify the morphology of the sacrum and sacroiliac joints, producing unequal relationships between the vertebral column and the two sides of the pelvis [36,37]. The structural relevance of these configurations depends on their symmetry, degree of fusion, articular relationships, and effects on adjacent vertebral segments [3,36,37]. Their presence alone does not establish that they are the primary cause of neurological or locomotor dysfunction [3,36,37].

The caudal boundary of the sacrum forms the transition between the fused sacral unit and the mobile caudal vertebral series. Transitional configurations at this level may involve mixed sacral and caudal characteristics or altered separation between the terminal sacral and first caudal segments. Developmentally, such configurations can be interpreted in relation to regional identity and posterior axial patterning [7,8,27].

The close developmental relationship between the sacral and caudal regions is particularly evident in Manx-associated phenotypes. In affected cats, reduction or absence of the tail may occur together with variable abnormalities of the sacral and caudal vertebrae and, in more extensive phenotypes, associated neural structures [18,19]. The external tail phenotype therefore does not necessarily indicate the anatomical extent of the underlying posterior axial disturbance [18,19].

From a developmental–pathological perspective, the sacral region contains two principal zones of vulnerability. At the lumbosacral junction, incomplete lumbar–sacral specification may alter vertebral incorporation and sacroiliac relationships [7,8,27,36,37]. At the sacrocaudal junction, disturbances of posterior axial development may affect the transition between the fused sacrum and the mobile caudal series [18,19]. The sacrum therefore represents both a fused anatomical structure and a developmental and biomechanical interface between the trunk, pelvis, and tail.

### 4.5. Caudal Region

The caudal region is the most numerically and morphologically variable part of the vertebral column in dogs and cats. Both species generally possess approximately 20 caudal vertebrae, with considerable variation among individuals and anatomical sources [1,2,32,33]. Unlike the relatively stable cervical, thoracic, lumbar, and sacral formulae, caudal vertebral number is not fixed and contributes, together with vertebral proportions and configuration, to variation in tail length and external morphology.

Caudal vertebrae undergo progressive structural simplification from the base toward the tip of the tail. Proximal caudal vertebrae retain a recognizable vertebral body, vertebral arch, spinous and transverse processes, and articular processes. More distally, the arches and processes gradually regress, and the terminal vertebrae may consist predominantly of small cylindrical vertebral bodies [1,2,32,33]. This proximodistal gradient reflects the transition from a structurally supported and muscularly controlled tail base to an increasingly mobile distal segment.

Intervertebral discs remain relatively well developed throughout much of the caudal series and contribute to mobility between adjacent segments. Proximal caudal vertebrae may also be associated with ventral haemal arches, commonly termed chevrons, or with paired haemal processes. The haemal arches may have a V- or Y-shaped configuration and protect the median caudal vessels while also providing attachment sites for caudal musculature [1,2,32,33].

The first caudal vertebra forms the mobile component of the sacrocaudal junction, and its morphology is therefore interpreted in relation to the terminal sacral segment [1,2,32,33]. Partial fusion, incomplete separation, or mixed sacral and caudal characteristics may indicate transitional morphology rather than isolated numerical variation. Developmentally, such configurations can be interpreted in relation to regional identity and posterior axial patterning [7,8,27]. Accurate classification therefore incorporates both the vertebral configuration and its structural relationship with the sacral apex.

Developmental abnormalities of the caudal region may affect vertebral number, segmentation, symmetry, fusion, or individual vertebral shape. Because the caudal series develops as part of the posterior body axis, extensive disturbances may involve not only the external tail but also the sacrum, vertebral canal, spinal cord, meninges, and associated neural structures [3,4,5,6,18,19,24]. The external appearance of the tail therefore provides only a partial indication of the anatomical extent of a posterior axial malformation [18,19].

Breed-associated caudal phenotypes further illustrate that similar external tail configurations may have distinct anatomical and genetic bases. Mutant *TBXT* alleles are associated with Manx short-tail phenotypes in cats [18,19] and with natural bobtail phenotypes in several dog breeds [20,21], although not all naturally short-tailed dogs share the same genetic basis [21]. In Bulldogs, French Bulldogs, and Boston Terriers, the *DVL2* frameshift variant contributes to the screw-tail morphological complex [16,17]. These associations reinforce the need to distinguish normal variation in caudal vertebral number from congenital reduction, intrinsic vertebral malformation, segmentation defects, fusion, and sacrocaudal dysgenesis.

From a developmental–pathological perspective, the caudal region therefore combines substantial normal anatomical variability with susceptibility to abnormalities of posterior axial development. Variation in caudal vertebral number is distinct from congenital reduction, vertebral deformation, segmentation defects, fusion, and sacrocaudal dysgenesis [3,18,19,20,21]. Externally similar tail phenotypes may therefore differ anatomically, developmentally, and genetically.

Together, the cervical, thoracic, lumbar, sacral, and caudal regions demonstrate that congenital vertebral anomalies cannot be interpreted independently of their axial location. Regional anatomy determines which vertebral components are affected and how an anomaly interacts with adjacent structures [1,2,32,33]. Developmental context is required to distinguish normal anatomical variation from altered regional identity or a broader disorder of axial formation [4,5,6,7,8,24,27]. The principal anatomical characteristics and developmental–pathological vulnerabilities of each vertebral region are summarized in Table 2.

## 5. Congenital and Breed-Associated Vertebral Anomalies in Dogs and Cats

Congenital and breed-associated vertebral anomalies in dogs and cats constitute a heterogeneous group of conditions ranging from minor anatomical variations to complex developmental malformations involving multiple vertebral components and adjacent neural structures [3,9,10,11]. Their developmental and pathological interpretation should not rely exclusively on the presence of an abnormal vertebral shape or number, because morphologically similar lesions may arise from distinct developmental mechanisms and may have markedly different structural and clinical consequences [3,9,10,11].

The significance of a vertebral anomaly depends on its axial location, the vertebral component affected, the degree and direction of deformation, the number and distribution of involved segments, and its structural relationship with the spinal cord, meninges, nerve roots, ribs, sacrum, or pelvis [3,9,10,11,36,37]. A localized and symmetrical anomaly may remain an incidental anatomical finding, whereas asymmetrical development, progressive angulation, vertebral canal narrowing, or involvement of a mechanically important junction may substantially increase its pathological relevance [3,9,10,11,36,37]. Vertebral morphology must therefore be interpreted within its regional anatomical context.

Axial transition zones, including the cervicothoracic, thoracolumbar, lumbosacral, and sacrocaudal junctions, require particular attention because they represent boundaries between vertebral regions with distinct structural identities [7,8,27,30,36,37]. Anomalies occurring at these levels may reflect incomplete specification of regional identity rather than a simple alteration in vertebral number [7,8,27,30]. By contrast, block vertebrae and complex congenital fusions are morphologically compatible with disturbances of segmentation or resegmentation [4,5,6,24], whereas wedge-shaped, butterfly-shaped, or asymmetrical vertebral bodies are compatible with abnormalities affecting the formation of specific vertebral components [3,4,5,6,9,10,11,24].

Breed-associated phenotypes introduce an additional level of complexity. Artificial selection and the genetic structure of domestic breeds can increase the frequency of distinctive conformational traits, including screw tails, natural bobtails, and other breed-specific axial morphologies [12,13,14,16,17,20,21]. The *DVL2*-associated phenotype in screw-tailed dog breeds and the *TBXT*-associated phenotypes in Manx cats and naturally bobtailed dogs represent well-characterized genetic associations in domestic carnivores [16,17,18,19,20,21]. However, neither mechanism should be generalized to all vertebral or caudal abnormalities with a similar external appearance, because comparable phenotypes may arise through distinct genetic and developmental mechanisms [16,17,18,19,20,21].

Accordingly, the following sections examine vertebral anomalies by region, integrating their morphological characteristics, developmentally plausible mechanisms, breed distribution, and relationships with adjacent neural and musculoskeletal structures. This regional framework facilitates distinction between normal anatomical variation and abnormalities of axial identity, segmentation or resegmentation, vertebral component formation, neural arch closure, and posterior axial development [4,5,6,7,8,18,19,20,21,24,27,31].

### 5.1. Atlas–Axis and Craniocervical Junction Anomalies

The craniocervical junction comprises the caudal occipital region, atlas, axis, their articular surfaces, and the ligamentous structures that stabilize the occipitoatlantal and atlantoaxial joints [1,32,33]. Congenital abnormalities may affect the occipital bone, atlas, axis, or their articulations individually or may occur as complex combinations involving several of these components [3,38,39,40,41,42,43,44]. Atlantoaxial instability may coexist with dens abnormalities and other craniocervical malformations and therefore represents a functional and biomechanical state associated with one or more structural abnormalities rather than a uniform morphological lesion [38,44].

Congenital atlantoaxial instability is characterized by abnormal mobility or alignment between the atlas and axis in association with developmental abnormalities of the dens, atlas, articular surfaces, supporting ligaments, or a combination of these structures [3]. It is reported most frequently in small- and toy-breed dogs, whereas feline cases are uncommon [3,38,44]. Congenital atlantoaxial instability is distinct from traumatic atlantoaxial luxation affecting an otherwise normally formed articulation, although trauma may precipitate clinical deterioration in an animal with a pre-existing structural abnormality [3,44].

The dens is a major osseous stabilizer of the atlantoaxial articulation [1,32,33]. Documented developmental abnormalities include absence or hypoplasia of the dens [3,44], reduced relative length and altered angulation [39], separation or a short, rounded configuration [38], and dens dysraphism [44]. Toy-breed dogs with atlantoaxial instability have been shown to have a lower dens-to-axis length ratio and a greater dens angle than nonaffected toy-breed dogs [39]. Atlantoaxial stability also depends on the transverse ligament of the atlas, the apical and alar ligaments, articular congruence, and the overall morphology of the atlas and axis [1,3,32,33].

Developmental abnormalities of the atlas commonly involve incomplete ossification or failure of fusion of its constituent components. Osseous defects may occur at the normal fusion sites between the paired components of the neural arch and the intercentrum and may affect the dorsal arch alone or both dorsal and ventral components [40,41]. Their structural significance varies. Incomplete ossification may occur without neurological signs when atlantoaxial subluxation is absent, whereas its association with atlantoaxial subluxation may contribute to abnormal alignment and reduced structural integrity [40,41]. In dogs with atlantoaxial instability, incomplete ossification of the dorsal neural arch and dens abnormalities are both common, although neither abnormality is present in every affected animal [42,43].

Atlas morphology also varies with body size. In small dogs, the dorsal neural arch is generally thinner and contains less trabecular bone than in larger dogs [40]. A thin, predominantly cortical dorsal arch with a structurally vulnerable midline region has also been documented in Maltese dogs [43]. These morphological features are distinct from discrete defects of incomplete ossification. Differentiation between morphological variation and incomplete ossification is based on osseous continuity and the configuration of the atlas components [40,43].

Broader craniocervical junction abnormalities may include atlantooccipital overlapping, occipitoatlantal synostosis or occipitalization of the atlas, indentation or deformation of the occipital bone, and complex combinations involving the dens, atlas, and atlantoaxial articulation [38,44]. These abnormalities may coexist with atlantoaxial instability and alter the spatial relationships between the foramen magnum, atlas, axis, and cranial cervical spinal cord [38,44]. Their pathological relevance depends on the degree of articular incongruence, malalignment, vertebral canal compromise, and involvement of adjacent neural structures rather than solely on the presence of an isolated morphological variation [3,38,44].

The morphological severity of a craniocervical anomaly does not always correspond directly to its functional significance. Incomplete ossification without atlantoaxial subluxation may remain an incidental structural finding, whereas instability may alter vertebral alignment and compromise the vertebral canal [3,40,41,44]. Assessment therefore incorporates the primary osseous abnormality, ligamentous competence, articular congruence, vertebral alignment, and relationships with adjacent neural structures [3,38,39,44]. Neurological abnormalities, when present, reflect the functional consequences of these structural relationships and are considered separately from the anatomical classification of the underlying lesion.

Craniocervical junction abnormalities are uncommon in cats [44]. Documented feline abnormalities include absence or dysraphism of the dens, incomplete ossification of the dorsal arch of the atlas, atlantoaxial instability, occipitoatlantal synostosis, and occipital bone deformation [44]. The coexistence of several osseous abnormalities in the same animal indicates that atlantoaxial displacement may represent only one component of a broader craniocervical malformation [44].

From a developmental–pathological perspective, abnormalities of this region can be classified according to the component principally affected: the dens and axis, the dorsal or ventral components of the atlas, the occipitoatlantal boundary, the atlantoaxial articulation, or a combination of these structures [38,40,41,42,43,44]. This component-based classification separates primary structural abnormalities involving vertebral formation, fusion, or ossification [4,5,6,24,40,41,42,43,44] from secondary instability, malalignment, vertebral canal compromise, and neural injury [3,38,39,40,41,42,43,44].

### 5.2. Cervical and Cervicothoracic Vertebral Anomalies

Apart from the specialized malformations of the atlas–axis complex, congenital abnormalities of the cervical region may involve vertebral number, regional identity, dorsal arch formation, segmentation, or the formation of individual vertebral components [3,4,5,6,7,8,24]. True numerical variation is uncommon because the seven-vertebra cervical formula is strongly conserved in mammals, including dogs and cats [1,2,28,29,32,33]. When C7 develops rib-bearing or other thoracic-like characteristics, the resulting morphology is consistent with altered cervicothoracic identity rather than a simple numerical anomaly [7,8,27,28,29,30].

#### 5.2.1. Cervicothoracic Transitional Vertebrae and Cervical Ribs

A cervicothoracic transitional vertebra is a congenital segment situated at the boundary between the cervical and thoracic regions that possesses morphological characteristics of both regions [30]. A common expression is partial thoracization of C7, manifested by unilateral or bilateral rib-like structures [7,8,27,30,45]. Developmentally, this phenotype is distinct from an abnormality of vertebral segmentation. Congenital fusion or incomplete separation of adjacent vertebrae is generally interpreted in relation to disturbances of segmentation or resegmentation [4,5,6,24]. By contrast, a transitional C7 retains its segmental individuality but exhibits characteristics normally associated with the thoracic region. A rib-bearing C7 is therefore morphologically consistent with a partial homeotic transformation toward thoracic identity [7,8,27,28,29,30].

In the comparative study by Brocal et al. [30], cervical ribs originated from C7 and occurred either unilaterally or bilaterally, with their morphology classified according to length (short or long) and the presence or absence of an articulation. Among the breeds examined by computed tomography, Pugs showed the highest frequency, with cervical ribs identified in 45 of 97 animals (46%), significantly more frequently than in the other breeds included in the study. Their presence was associated with morphological changes of the C7 dorsal spinous process, including higher C7/T1 spinous-process ratios, supporting interpretation of the rib-bearing C7 as a partial homeotic transformation toward thoracic identity, although altered *HOX* gene expression was not directly investigated. Within the computed tomographic cohort of Pugs, cervical ribs were also associated with thoracolumbar transitional vertebrae, although this relationship was not reproduced in the radiographic subset, and affected animals were more likely to possess 26 rather than 27 presacral vertebrae [30]. No significant associations were identified with vertebral body formation defects, bifid spinous processes, caudal articular process hypoplasia or aplasia, or abnormal sacral morphology [30]. These findings indicate that cervical ribs did not occur as part of a uniform constellation of vertebral abnormalities in the examined Pugs.

Cervical ribs were also identified in Pugs without clinical signs attributable to congenital vertebral malformations [30]. Their presence may therefore represent a clinically silent morphological phenotype and does not independently establish pathological significance. The study did not directly assess neurovascular compression attributable to cervical ribs, and the potential neurovascular consequences of this phenotype in individual dogs therefore remain unresolved [30].

Cervical numerical and regional variation has also been documented in Japanese Bobtail cats. Reported findings included cats with six rather than seven cervical vertebrae, cervicothoracic transitional regions characterized by ribs on C7, and other anomalous ribs [45]. These findings occurred within a broader spectrum of abnormal vertebral formulae and caudal vertebral malformations. Within the examined pedigree-derived population, abnormal vertebral formulae and the position of the caudal kink did not coincide with morbidity or mortality [45]. Because these findings were obtained from a small pedigree-derived population selected for the Japanese Bobtail phenotype, they characterize the examined cohort and do not establish a shared developmental mechanism for the different abnormalities.

From a developmental–pathological perspective, a rib-bearing C7 is morphologically consistent with altered regional identity at the cervicothoracic boundary [7,8,27,28,29,30]. Morphological characterization includes whether the anomaly is unilateral or bilateral, the length and articular configuration of the rib-like structure, and the presence of associated changes in presacral vertebral number or at other axial transition zones. This component-based assessment distinguishes transitional morphology from congenital vertebral fusion without assigning a molecular mechanism that has not been directly demonstrated.

#### 5.2.2. Cervical Dorsal Arch Defects and Spinal Dysraphism

Spina bifida is a congenital vertebral malformation defined anatomically by incomplete formation or fusion of the dorsal vertebral arches [46,47]. The osseous defect may occur without external protrusion of the meninges or spinal cord, corresponding to a closed or occult form, or may be associated with protrusion of the meninges alone, as a meningocele, or of the meninges together with neural tissue, as a meningomyelocele [46,47]. Spinal dysraphism represents a broader developmental category that may include associated abnormalities of the spinal cord, meninges, and cutaneous midline structures [46,48].

An isolated bifid spinous process does not, by itself, establish the presence of spina bifida. Such a configuration may represent localized abnormal formation of the dorsal process without evidence of an extensive vertebral arch defect or associated meningeal and neural involvement [30]. Accordingly, anatomical terminology can distinguish a divided spinous process from spina bifida and broader spinal dysraphism according to the structures involved.

In the study by Brocal et al. [30], bifid spinous processes were identified in approximately one-third of the 130 Pugs examined and were located predominantly at T1; one dog had bifid spinous processes at C5 and C6, and another had the abnormality at T5. These findings were reported without myelomeningocele, and no significant association was identified between bifid spinous processes and cervical ribs [30]. In this context, the bifid spinous process represents a localized dorsal process abnormality rather than, by itself, evidence of spina bifida.

Cervical or cervicothoracic spina bifida has been reported sporadically in dogs [46,47,48]. In a series of three affected animals, Arias et al. [47] described one dog with closed spina bifida involving the caudal cervical and cranial thoracic vertebrae together with open spina bifida of the sacrocaudal region. This case demonstrates that dorsal arch closure defects may affect multiple discontinuous regions and may differ in morphological expression within the same animal.

A localized cervical phenotype was subsequently reported in a Labrador Retriever mixed-breed dog with spina bifida at C3 associated with type I and type IV dermoid sinuses [48]. Computed tomography demonstrated incomplete dorsal closure of C3, whereas magnetic resonance imaging characterized the associated sinus tracts and adjacent neural and soft-tissue structures [48]. This association demonstrates that cervical spina bifida may occur as part of a broader midline dysraphic disorder involving both vertebral and cutaneous components.

The pathological significance of a cervical dorsal arch defect depends on its extent and on the presence of associated meningeal, neural, or cutaneous abnormalities. Closed forms may remain clinically silent, whereas meningocele, meningomyelocele, or a dermoid tract extending toward the vertebral canal may be associated with neurological dysfunction [46,47,48]. Anatomical assessment therefore separates the osseous defect from associated meningeal, spinal cord, and soft-tissue lesions. Computed tomography is particularly useful for defining the location and osseous extent of a dorsal arch defect, whereas magnetic resonance imaging provides complementary information when involvement of the spinal cord, meninges, dermoid tracts, or other soft tissues is suspected [46,48]. The terms bifid spinous process, incomplete dorsal arch formation, spina bifida occulta, meningocele, meningomyelocele, and dermoid sinus therefore refer to distinct anatomical configurations defined by the structures involved.

From a developmental–pathological perspective, cervical dorsal arch abnormalities may be differentiated according to the extent of the osseous defect and the presence or absence of associated meningeal, neural, and cutaneous involvement [46,47,48]. This component-based interpretation distinguishes an isolated bifid spinous process from true spina bifida and from a broader spinal dysraphism. Developmentally, these abnormalities are compatible with disturbed formation or fusion of the dorsal vertebral components, although the precise mechanism may vary among individual phenotypes [4,5,6,24].

#### 5.2.3. Cervical Block Vertebrae and Congenital Vertebral Fusion

Cervical block vertebrae are congenital malformations characterized by partial or complete fusion of two or more adjacent vertebral segments. The fusion may involve the vertebral bodies, dorsal laminae, articular facets, or combinations of these components [3,49,50,51]. Developmentally, this morphology is consistent with disturbance of normal segmentation or resegmentation and failure of separation between adjacent vertebral primordia [4,5,6,24].

A block vertebra is anatomically distinct from a transitional vertebra. In transitional morphology, an individual vertebra retains its segmental separation but acquires characteristics of an adjacent axial region. In a block vertebra, the principal abnormality is partial or complete loss of the normal anatomical boundary between consecutive vertebral segments. Congenital fusion is also anatomically distinct from acquired ankylosis or proliferative osseous bridging. Morphological assessment includes the configuration of the vertebral bodies and arches, the presence and morphology of the intervening disc space, the pattern of osseous continuity, and the condition of adjacent segments [3,49].

Fernandes et al. [49] documented several patterns of congenital cervical fusion in a series of nine dogs with cervical vertebral malformations. Two young dogs exhibited complex, multilevel abnormalities described as Klippel–Feil-like. In one dog, C3 and C4 were fused asymmetrically in association with malformations of the atlas and axis. In the second, partial fusion involved the caudal body of C2, the hypoplastic body of C3, and C4, together with incomplete formation of the C4–C5 intervertebral disc and fusion of the dorsal laminae [49]. Two additional dogs had more localized congenital cervical fusions, involving C4–C5 and C2–C3, respectively. These observations demonstrate that congenital cervical fusion may occur either as a localized two-segment abnormality or as part of a more extensive multicomponent pattern.

In the two dogs with localized congenital fusion described by Fernandes et al. [49], intervertebral disc disease occurred immediately caudal to the fused segment. The authors interpreted these findings as adjacent-segment disease potentially associated with increased mechanical stress at mobile levels bordering a rigid congenital fusion [49]. This proposed association was based on two dogs within a small retrospective series and therefore provides limited evidence for a general relationship between congenital cervical fusion and adjacent-segment disease.

In a radiographic study of 73 French Bulldogs, Kuricová et al. [50] identified five block vertebrae across the vertebral column, including one cervical fusion involving C2 and C3. Vertebral body malformations were substantially more frequent than block vertebrae, and no statistically significant predisposition for a particular abnormal vertebral level was demonstrated [50]. The isolated C2–C3 case documents cervical block vertebra formation in a French Bulldog without providing evidence of a breed-specific predisposition to cervical fusion. The study population comprised 73 French Bulldogs and contained substantially more thoracic than cervical abnormalities overall.

More extensive cervical fusion was reported by Lin and Coolman [51] in two dogs with block vertebrae extending from C2 to C5 and from C2 to C4, respectively. Both dogs also developed traumatic atlantoaxial subluxation. The authors hypothesized that the long, rigid fused cervical segment produced a “fulcrum effect” at the mobile atlantoaxial joint and increased susceptibility to traumatic displacement [51]. This proposed relationship is anatomically plausible but remains a hypothesis based on two clinical cases rather than evidence of a general causal association.

The pathological significance of a cervical block vertebra depends on the number of fused segments, the symmetry and orientation of the fusion, vertebral canal dimensions, spinal alignment, and the condition of adjacent mobile segments [3,49,51]. A well-aligned congenital fusion may remain an incidental finding, whereas asymmetrical or multilevel fusion may be associated with angular deformation, vertebral canal compromise, altered mechanical loading, or degeneration of adjacent intervertebral discs [3,49]. Neurological manifestations, when present, may reflect the structural and biomechanical consequences of the fusion rather than defining the morphology of the block vertebra itself.

From a developmental–pathological perspective, cervical block vertebrae can be characterized according to the vertebral levels involved, the number and continuity of fused segments, the vertebral components affected, the morphology of the intervening disc space, and the presence of associated axial malalignment or adjacent-segment abnormalities [49,50,51]. This component-based interpretation distinguishes a primary segmentation or separation defect from a transitional vertebra and from secondary degenerative or instability-related changes [4,5,6,24,49,50,51].

#### 5.2.4. Cervical Vertebral Body Formation and Ossification Defects

Congenital abnormalities affecting the cervical vertebral bodies include partial hypoplasia, asymmetrical development, wedge-shaped configuration, incomplete formation, and incomplete ossification of individual vertebral components [3,50,52,53]. These abnormalities have sometimes been included within the broad category of hemivertebrae in veterinary literature, although their morphology varies according to the vertebral component affected and the direction and extent of the formation defect. A component-based description provides greater anatomical specificity when the abnormal configuration can be defined [3,9,10,11,52].

Cervical vertebral body malformations are less commonly documented than thoracic vertebral body malformations, particularly in brachycephalic screw-tailed dogs, but may have substantial structural consequences when they alter vertebral alignment or vertebral canal dimensions [3,9,10,11,50,52]. In the series of five dogs evaluated by Besalti et al. [52] using the Nasca classification, a Doberman Pinscher had a single type 2a wedge-shaped vertebra at C7, whereas a mixed-breed dog had multiple type 4a abnormalities extending from C7 to T7. Both dogs presented with nonambulatory tetraparesis [52]. These observations document severe neurological dysfunction in association with cervical and cervicothoracic vertebral body malformations, but they do not define the clinical significance of individual morphological subtypes beyond the reported cases.

Cervical vertebral abnormalities have also been documented in French Bulldogs. In a radiographic study of 73 dogs, Kuricová et al. [50] identified 13 cervical abnormalities among 67 abnormal vertebrae recorded throughout the vertebral column. Eight cervical abnormalities were classified as hemivertebrae and involved C5, C6, or C7; the remaining cervical findings included atlantoaxial instability and a C2–C3 block vertebra. No statistically significant predisposition for a particular abnormal vertebral level was identified [50]. These findings confirm that vertebral body malformations may occur in the caudal cervical region of French Bulldogs, although thoracic abnormalities were considerably more frequent in the same study population.

A distinct developmental phenotype has been reported in cats. Dean et al. [53] described a 21-week-old Domestic Shorthair cat with incomplete ossification of C2 and C3 demonstrated by computed tomography. The cat presented with acute-onset severe nonambulatory tetraparesis and a C1–C5 neuroanatomical localization. Following conservative management, normal ambulation was regained within four weeks, although some reluctance to jump persisted [53]. This case documents incomplete cervical ossification in association with neurological dysfunction but does not, on its own, establish the precise biomechanical mechanism linking the structural abnormality to the clinical presentation. The phenotype is morphologically distinct from a wedge-shaped or unilateral vertebral body formation defect.

The pathological relevance of a cervical vertebral body abnormality depends on the vertebral component affected, the degree and direction of deformation, symmetry, vertebral level, spinal alignment, vertebral canal dimensions, and relationships with adjacent intervertebral discs and articular structures [3,11,52,53]. Minor, well-aligned abnormalities may remain clinically silent, whereas displacement, marked asymmetry, multilevel involvement, or vertebral canal narrowing may increase the likelihood of structural or neurological consequences [3,52,53]. Neurological manifestations therefore reflect the structural effects associated with the malformation rather than defining its morphological category.

From a developmental–pathological perspective, cervical vertebral body formation defects are distinct from congenital vertebral fusion, transitional morphology, and abnormalities affecting primarily the ossification process. Abnormal shape or incomplete formation of the vertebral body is developmentally compatible with disturbance of formation of the centrum or its cartilaginous template [4,5,6,24]. Congenital fusion between adjacent segments is more closely related to disturbances of segmentation, resegmentation, or vertebral separation [4,5,6,24], whereas incomplete ossification represents a distinct morphological pattern in which vertebral elements are present but are not fully ossified [53]. Accordingly, a component-based classification integrates conventional terminology with a detailed description of the vertebral component and structural configuration demonstrated by imaging.

### 5.3. Thoracic Vertebral Anomalies

Thoracic vertebral malformations are commonly documented in brachycephalic screw-tailed dogs, particularly French Bulldogs, English Bulldogs, Pugs, and Boston Terriers [9,10,11]. Documented abnormalities at this level include variation in vertebral number, transitional morphology, vertebral body formation defects, congenital fusion, dorsal arch and spinous process abnormalities, and dysplasia or aplasia of the articular processes [9,10,11]. The high frequency of thoracic vertebral abnormalities does not imply that every affected animal develops neurological disease. Congenital thoracic malformations are commonly identified in dogs examined for reasons unrelated to spinal dysfunction, and affected animals may possess one or more abnormal vertebrae without corresponding neurological signs [10,11]. Their pathological significance depends on the type and distribution of the abnormalities and on whether they alter spinal alignment, vertebral canal dimensions, segmental stability, or the relationship between the vertebral column and the spinal cord [3,9,10,11].

The thoracic region has a distinctive anatomical context because each vertebra is structurally integrated with the ribs and thoracic cage [1,2,32,33]. Consequently, asymmetrical development of a thoracic vertebra may influence vertebral column alignment, rib orientation, and thoracic symmetry. The direction and extent of vertebral body deficiency are particularly relevant because ventral, lateral, or combined asymmetrical defects may be associated with different patterns of kyphotic, scoliotic, or combined angular deformation [9,11].

Breed-associated differences are evident in both the frequency and morphology of thoracic anomalies. In the populations studied, French Bulldogs and English Bulldogs frequently exhibited vertebral body formation defects, whereas Pugs showed a broader spectrum that included transitional vertebrae, dorsal process abnormalities, articular process abnormalities, and vertebral body malformations [10,11]. These differences indicate that thoracic abnormalities occurring in brachycephalic breeds should not be regarded as a single uniform phenotype.

Although the *DVL2* frameshift variant contributes to the broader axial phenotype of Bulldogs, French Bulldogs, and Boston Terriers, it does not provide a universal explanation for all thoracic vertebral abnormalities occurring in these or other breeds [16,17]. Thoracic vertebral morphology should therefore be classified according to its anatomical characteristics before a specific developmental or genetic mechanism is assigned.

Accordingly, the following subsections examine thoracic anomalies according to the principal anatomical disturbance: vertebral number and regional identity, vertebral body formation, segmentation and congenital fusion, dorsal arch formation, articular process development, and associated rib morphology. This organization distinguishes the primary structural abnormality from secondary spinal curvature, instability, or neural compromise.

#### 5.3.1. Thoracic Vertebral Number and Thoracolumbar Transitional Morphology

Dogs and cats normally possess 13 thoracic vertebrae, whose regional identification is based principally on their relationship with the ribs and associated costal structures [1,2,32,33]. Reported congenital variation includes regional counts of 12 or 14 thoracic vertebrae, as well as vertebrae with mixed regional morphology at the cervicothoracic or thoracolumbar boundary [10,45,50,54]. Thoracic vertebral count should therefore be interpreted together with the anatomical characteristics of the boundary segments rather than as an isolated numerical observation.

A thoracolumbar transitional vertebra combines thoracic and lumbar characteristics. Lumbarization of T13 may be expressed by absence or hypoplasia of one or both associated ribs and modification of the transverse processes toward lumbar morphology. Conversely, thoracization of L1 may be characterized by unilateral or bilateral ribs or rib-like costal processes arising from the first lumbar segment [10,54]. This mixed morphology is anatomically distinct from a block vertebra because the intervertebral boundary between adjacent vertebral segments is preserved [10]. Developmentally, such morphology is compatible with alteration of regional axial identity [7,8,27,29]. However, the imaging studies considered here did not investigate the underlying molecular mechanism [10,45,50,54].

Marked breed-associated differences were documented by Ryan et al. [10] in neurologically normal brachycephalic dogs examined by computed tomography. Seventeen of 68 Pugs (25.0%) had only 12 thoracic vertebrae, whereas 21 of 68 (30.9%) had a transitional T13 displaying lumbar characteristics. All 62 French Bulldogs had 13 thoracic vertebrae, although three dogs (4.8%) had a transitional T13. All 41 English Bulldogs also had 13 thoracic vertebrae; four dogs (9.8%) had transitional morphology, comprising three animals with lumbar characteristics of T13 and one with cervical characteristics of T1 [10].

Numerical variation was also identified radiographically in French Bulldogs by Kuricová et al. [50]. Among 73 dogs with radiographs extending from C1 through S3, three had a 14th thoracic vertebra and one had only 12 thoracic vertebrae. Variation in lumbar number was also documented in the same cohort, with eight lumbar vertebrae in five dogs and six lumbar vertebrae in two dogs [50]. The occurrence of variation in both thoracic and lumbar regional counts within this population illustrates the importance of considering regional counts in relation to the complete presacral vertebral formula [50].

Thoracolumbar transitional morphology has also been documented in cats. In a retrospective radiographic study of 200 cats, Newitt et al. [54] identified congenital abnormalities in 46 animals, involving 54 spinal segments. Transitional abnormalities were the most frequent category, affecting 51 spinal segments, and thoracization of L1 was identified in 18 cats. The additional ribs arising from L1 were often small or fragmented and could potentially be confused radiographically with rib fractures [54]. Recognition of these variants is therefore important for correct vertebral enumeration and anatomical localization.

Thoracic numerical variation was also prominent in cats expressing the Japanese Bobtail kinked-tail phenotype. Fourteen of 17 kink-tailed cats in which the complete vertebral column was evaluated radiographically had a formula containing 12 rather than 13 thoracic vertebrae, most commonly C7–T12–L7–S3 [45]. One cat also had a thoracolumbar transitional vertebra characterized by a rib arising from L1. Within the examined pedigree-derived cohort, neither the abnormal vertebral formula nor the position of the caudal kink coincided with morbidity or mortality [45]. These observations characterize a small, phenotype-selected cohort and do not provide population-level estimates for the wider feline population.

In the populations examined, thoracic numerical variation was documented primarily as an anatomical and diagnostic finding rather than as a demonstrated cause of neurological disease [10,45,54]. Numerical or transitional variation may affect rib counting, vertebral enumeration, and anatomical localization during imaging or surgical planning [10,45,54]. Small, unilateral, or fragmented ribs associated with a transitional segment may additionally complicate differentiation between congenital morphology and acquired rib injury [54].

At a descriptive developmental level, an atypical thoracic count may reflect either variation in the overall presacral vertebral formula or altered regional identity of a vertebra situated at an axial boundary [7,8,27,29]. The available imaging evidence does not permit attribution of these phenotypes to a specific molecular or embryological mechanism in individual dogs or cats. Anatomically based classification integrates whole-column enumeration with evaluation of the ribs, transverse processes, intervertebral spaces, and morphology of the adjacent vertebral regions [10,45,50,54]. This approach separates numerical and transitional variation from vertebral body formation defects and congenital fusion.

#### 5.3.2. Thoracic Vertebral Body Formation Defects

Thoracic vertebral body formation defects are characterized by partial absence or reduced development of one or more regions of the vertebral body while the intervertebral boundaries with adjacent segments remain identifiable [3,9,11]. They are therefore anatomically distinct from block vertebrae, in which segmentation between adjacent vertebrae is absent or incomplete [3,9,11]. Because the specific molecular or embryological mechanism cannot usually be determined from postnatal imaging alone, these abnormalities can be classified according to the location, direction, and extent of the deficient vertebral body tissue [9,11].

The principal configurations include ventral aplasia, traditionally termed a dorsal hemivertebra; ventral hypoplasia, producing a ventral wedge-shaped vertebra; lateral aplasia or hypoplasia; ventrolateral aplasia, traditionally termed a dorsolateral hemivertebra; ventrolateral hypoplasia; ventral and median aplasia, producing a butterfly vertebra; ventral and median hypoplasia; and symmetrical hypoplasia, producing a shortened vertebral body [9,55]. More recent imaging studies have additionally described a dorsal wedge-shaped configuration, characterized by reduced height of the dorsal portion of the vertebral body [15,56]. The term hemivertebra has been applied variably across the veterinary literature, sometimes specifically to directional aplasia and sometimes collectively to several vertebral body formation defects [9,10,55]. The use of an anatomical descriptor together with, where appropriate, the conventional term therefore provides greater morphological precision.

The structural effect of a formation defect depends on both its direction and severity. Ventral deficiency may be associated with focal kyphotic angulation, whereas lateral or ventrolateral deficiency may accompany scoliosis or combined angular deformation [9,55]. Symmetrical hypoplasia or a relatively symmetrical median defect may shorten the vertebral body without producing an equivalent degree of angular deformation. The final spinal configuration, however, also depends on the morphology of adjacent vertebrae, the number and distribution of affected segments, intervertebral alignment, and the presence of vertebral displacement or canal narrowing [15,55]. The descriptive shape of an individual vertebra does not, by itself, determine its pathological significance.

In the radiographic classification study by Gutierrez-Quintana et al. [9], 28 brachycephalic screw-tailed dogs with at least one congenital thoracic vertebral malformation were examined. Malformations affected 85 of 362 thoracic vertebrae (23.5%), and vertebral body formation defects constituted the most frequently identified category. Butterfly vertebrae accounted for 6.6% of the examined thoracic vertebrae, ventral wedge-shaped vertebrae for 5.5%, dorsal hemivertebrae for 0.8%, and dorsolateral hemivertebrae for 0.5% [9]. T7 was affected in 11 of the 28 dogs, followed by T8 and T12, each affected in eight dogs.

Breed-associated differences were clearly demonstrated by Ryan et al. [10] in 171 neurologically normal dogs examined by computed tomography. One or more vertebral body formation defects, collectively classified in that study as hemivertebrae, were identified in 58 of 62 French Bulldogs (93.5%), 30 of 41 English Bulldogs (73.2%), and 12 of 68 Pugs (17.6%) [10]. Multiple abnormalities predominated in the French and English Bulldog groups, and the regional distribution of affected vertebrae also differed among breeds. These findings demonstrate that both frequency and anatomical distribution of thoracic vertebral body malformations vary substantially among brachycephalic breeds.

De Decker et al. [55] further demonstrated breed-associated differences in vertebral body morphology in a computed tomography (CT) study of 160 French Bulldogs, Pugs, and English Bulldogs, all with at least one thoracic hemivertebra. Among the 120 neurologically unaffected dogs, 411 thoracic malformations were identified, with a median of three affected vertebrae per dog. Ventral and median hypoplasia was the most frequent subtype, followed by ventral and median aplasia and symmetrical hypoplasia [55]. Ventral aplasia, ventral hypoplasia, and symmetrical hypoplasia were associated with Pugs, whereas all lateral aplasia defects in the examined population occurred in French Bulldogs [55]. These breed–subtype associations are specific to the populations examined and do not establish universal breed-specific patterns.

Brown et al. [56] similarly demonstrated breed-associated morphological differences in an Australian cohort of French Bulldogs and Pugs. Butterfly morphology was associated with French Bulldogs, whereas transitional abnormalities were associated with Pugs; the authors also described a dorsal wedge-shaped subtype in 12 cases [56]. More recently, Moses et al. [57] reported that ventral hypoplasia was the most frequent abnormality in French and British Bulldogs, whereas symmetrical hypoplasia predominated in Pugs. These studies used related but nonidentical classifications and differed in study population, imaging modality, inclusion criteria, and classification method, limiting direct comparison of subtype frequencies.

Thoracic vertebral body formation defects are frequently present without corresponding neurological dysfunction. In Ryan et al. [10], most neurologically normal French and English Bulldogs had one or more affected thoracic vertebrae. Likewise, among the neurologically unaffected dogs examined by De Decker et al. [55], multiple abnormalities were common. Neither the presence of a malformed vertebra nor a greater number of affected vertebrae therefore establishes clinical relevance by itself [10,55,56].

Certain configurations and secondary structural changes are nevertheless associated with greater pathological significance. In De Decker et al. [55], ventral aplasia was associated with the greatest kyphotic angulation and the smallest remaining vertebral canal area among the evaluated subtypes. In the multivariable model, Pug breed, greater kyphotic angulation, fewer rather than more hemivertebrae, and ventrolateral hypoplasia were independently associated with neurological signs [55]. Structural severity of an individual morphological subtype and its independent statistical association with clinical status represent distinct findings.

In a small case series, Moissonnier et al. [58] compared neurologically normal French Bulldogs with four dogs affected by compressive spinal cord disease associated with thoracic hemivertebrae. The clinically affected dogs showed greater kyphotic angulation and vertebral displacement [58]. In the prospective study by Lackmann et al. [15], neurological deficits were associated with ventrolateral wedge-shaped and dorsolateral hemivertebral morphology, a kyphotic Cobb angle greater than 30°, and a vertebral step of at least 1.75 mm [15]. These cohort-specific measurements indicate that asymmetric vertebral body deficiency, focal angulation, displacement, and vertebral canal compromise may be more informative for assessing pathological significance than vertebral shape alone.

Published feline data on thoracic vertebral body formation defects are limited. The frameshift deletion in *DVL2* identified in Bulldogs, French Bulldogs, and Boston Terriers showed a strong relationship with the broader screw-tail phenotype, but thoracic vertebral abnormalities demonstrated variable expression among breeds [16,17]. In the imaging component of Niskanen et al. [17], homozygous American Staffordshire Terriers showed a clear association with caudal vertebral malformations, whereas thoracic or lumbar vertebral malformations were not identified in the examined dogs. The available evidence therefore supports *DVL2* as a contributor to a broader breed-associated axial phenotype but does not establish it as the direct cause of the number, distribution, or morphological diversity of thoracic vertebral body formation defects [16,17].

Thoracic vertebral body formation defects appear to be less frequently documented in cats within the literature reviewed. In the retrospective radiographic study of 200 cats by Newitt et al. [54], no hemivertebrae were identified, although transitional and block vertebrae were recorded. Tremolada et al. [59] examined six cats with persistent right aortic arch, four of which had at least one associated axial skeletal malformation. Two thoracic butterfly vertebrae, located at T8 and T12, were identified in one cat [59]. Because this was a very small, clinically selected series containing multiple concurrent abnormalities, it provides evidence that thoracic vertebral body formation defects can occur in cats but does not establish their prevalence in the wider feline population or a causal relationship with the vascular anomaly.

Thoracic vertebral body formation defects can be characterized according to the deficient portion of the vertebral body, the distinction between aplasia and hypoplasia, the affected vertebral level, and whether the abnormality is isolated or distributed across several segments. Anatomical assessment also incorporates spinal curvature, intervertebral alignment, vertebral canal dimensions, and the relationship of the malformed segment to adjacent ribs and vertebrae [9,10,15,55,56,57,58]. This component-based approach separates the primary formation defect from associated kyphosis, scoliosis, subluxation, instability, or spinal cord compromise without assigning a specific genetic or developmental mechanism in the absence of direct evidence.

#### 5.3.3. Thoracic Block Vertebrae

Thoracic block vertebrae are congenital abnormalities characterized by partial or complete fusion of two or more adjacent vertebral segments, with corresponding partial or complete loss of the normal intervertebral separation and disc space [3,9,10,60]. Fusion may involve two adjacent vertebrae or extend across several consecutive segments [3,9,60]. In complex malformations, non-segmentation of the vertebral bodies may coexist with separately formed vertebral arches [61] or with additional vertebral body formation defects [9,61]. Fusion confined to the dorsal spinous processes represents a distinct morphological category from block vertebrae and was recorded separately by Ryan et al. [10].

In veterinary morphological classifications, block vertebrae are categorized as segmentation defects [9,10]. Diagnostic imaging defines the vertebral components involved and can demonstrate whether fusion occurs alone or together with vertebral body formation abnormalities, as documented in complex malformations combining segmentation and formation defects [9,61].

Clinical and structural expression varies among reported cases. Newitt et al. [54] identified block vertebrae in cats without associated clinical signs despite marked scoliosis, whereas Crowe et al. [60] described multiple thoracic block vertebrae with lordosis and adjacent intervertebral disc disease in a neurologically affected cat, and Couto et al. [61] documented combined segmentation–formation defects associated with marked angular deformation, vertebral canal stenosis, and spinal cord compression.

Ryan et al. [10] documented thoracic block vertebrae in neurologically normal brachycephalic dogs examined by computed tomography. Four of 62 French Bulldogs (6.5%) had block vertebrae, with six affected vertebral pairs comprising four occurrences at T11–T12 and two at T12–T13. Four of 41 English Bulldogs (9.8%) were also affected, with three block vertebrae at T11–T12 and one at T12–T13. No block vertebrae were identified in the 68 Pugs examined [10]. Although the affected French and English Bulldogs showed a predominantly caudal thoracic distribution, breed did not have a statistically significant influence on the prevalence of block vertebrae in this cohort (*p* = 0.086) [10].

The block vertebrae identified in the neurologically normal population of Ryan et al. [10] were not associated with neurological abnormalities referable to the vertebral column. In the separate group of ten dogs in which a thoracic vertebral malformation was considered responsible for neurological dysfunction, the abnormalities implicated were vertebral body formation defects rather than block vertebrae [10]. In this study, block vertebrae occurred in the neurologically normal cohort, whereas the thoracic vertebral malformations considered responsible for neurological dysfunction in the affected cohort were vertebral body formation defects [10].

Feline data are more limited. In the retrospective radiographic study of 200 cats by Newitt et al. [54], block vertebrae were identified in three animals. Despite marked associated scoliosis, the three affected cats had no clinical signs or palpable deformities attributable to the vertebral abnormalities [54].

A clinically significant combination of multiple thoracic block vertebrae and adjacent spinal pathology was described by Crowe et al. [60] in a 6-month-old Domestic Shorthair cat. The cat had 12 thoracic vertebrae, fusion of the T1–T3 vertebral bodies, and additional partial fusion at T4–T5 and T10–T11, together with focal cranial thoracic lordosis. Magnetic resonance imaging demonstrated T3–T4 intervertebral disc protrusion with severe spinal cord compression and generalized cervical and thoracic intervertebral disc degeneration [60]. The authors considered angular deformation (lordosis), abnormal loading of adjacent vertebrae, and adjacent-segment disease as probable contributors to the clinically significant intervertebral disc degeneration and protrusion [60].

Clinically important combined segmentation and formation defects have also been documented in dogs outside the brachycephalic screw-tailed breeds. Couto et al. [61] described three adult large-breed dogs with progressive thoracolumbar myelopathy associated with congenital vertebral malformations combining failures of vertebral body segmentation and formation. The abnormalities involved T13–L1, T8–T9, and T10–T11 and included marked kyphosis or kyphoscoliosis, vertebral canal narrowing, and spinal cord compression [61].

Thoracic block vertebrae can be characterized by the exact vertebral levels involved, the number of fused segments, the completeness and symmetry of fusion, the presence or absence of a residual intervertebral boundary, and the vertebral components involved. Associated vertebral body formation defects, spinal curvature, vertebral canal narrowing, and abnormalities of adjacent intervertebral discs provide additional anatomical context [10,54,60,61]. These features distinguish vertebral-body segmentation abnormalities from isolated fusion of dorsal spinous processes and from complex malformations combining segmentation and formation defects.

#### 5.3.4. Thoracic Dorsal Arch Defects and Spinal Dysraphism

Thoracic dorsal arch abnormalities include clefts or bifid morphology involving the dorsal arch or spinous process of an individual vertebra and fusion between the dorsal spinous processes of adjacent vertebrae [10,30]. Ryan et al. [10] recorded spina bifida and fused spinous processes as separate morphological categories, whereas Brocal et al. [30] separately recorded bifid spinous processes in Pugs. A bifid spinous process describes a divided spinous-process morphology within an individual vertebra, whereas fused spinous processes involve osseous continuity between the dorsal processes of adjacent vertebrae; neither term is equivalent to vertebral-body block formation [10,30].

Spina bifida is characterized anatomically by incomplete closure of one or more vertebral arches over the spinal canal [3,10,46]. The osseous defect may be skin-covered in closed forms or may occur together with abnormalities of the meninges, spinal cord, or other midline structures [46,62]. In open forms, meningeal or neural tissue may protrude through the vertebral arch defect [46]. Spinal dysraphism is a broader term for malformations involving structures related to the spinal midline and may occur with or without spina bifida [46,62,63].

Ryan et al. [10] documented marked breed-associated differences in thoracic dorsal arch morphology among neurologically normal brachycephalic dogs examined by computed tomography. Twenty-six of 68 Pugs (38.2%) had an abnormality classified in that study as spina bifida, exclusively at T1. One of 41 English Bulldogs (2.4%) had spina bifida at T10. Among 62 French Bulldogs, no animal had isolated spina bifida, although one vertebra showed spina bifida in combination with a vertebral body formation defect [10]. Pugs were significantly more frequently affected than the other breeds (*p* < 0.0001) [10]. In that study, spina bifida was defined radiographically as incomplete closure of the vertebral arches producing a cleft through the dorsal spinous process [10].

Brocal et al. [30] subsequently described bifid spinous processes as a separate morphological category in Pugs. Forty-nine bifid spinous processes were identified in 47 of 130 Pugs (36%), with T1 representing the predominant location; one Pug had bifid spinous processes at C5 and C6, and another had a bifid spinous process at T5 [30]. No myelomeningocele accompanied these findings, and bifid spinous processes were not associated with cervical ribs [30]. Ryan et al. [10] used the term spina bifida for incomplete closure of the vertebral arches with a cleft through the dorsal spinous process, whereas Brocal et al. [30] recorded bifid spinous process as a separate morphological variable. Both datasets show a predominance of dorsal midline abnormalities at T1 in Pugs, but the reported prevalence values are not directly interchangeable because the classification terms differ.

Fusion of adjacent dorsal spinous processes showed a different breed distribution in Ryan et al. [10]. This configuration was recorded in 14 of 62 French Bulldogs (22.6%) and eight of 41 English Bulldogs (19.5%), but in none of the 68 Pugs [10]. In Ryan et al. [10], fused spinous processes were recorded separately from block vertebrae, representing a distinct radiographic category rather than evidence of vertebral-body fusion.

In the populations examined by Ryan et al. [10], the frequent T1 dorsal arch defects observed in Pugs were not identified as the cause of neurological dysfunction. Three of the Pugs in the clinically affected group also had T1 spina bifida, but these findings were considered incidental; vertebral body formation defects were identified as the cause of spinal dysfunction in all ten clinically affected dogs [10]. Thus, within this cohort, the presence of a limited T1 dorsal arch cleft was insufficient to establish clinically significant spinal disease.

More extensive vertebral arch defects may coexist with abnormalities of the spinal cord and other midline structures. Wilson et al. [62] described four English Bulldogs and one Collie with spina bifida, in all of which clinical signs and morphological spinal cord abnormalities also fulfilled the authors’ criteria for spinal dysraphism. Urinary and fecal incontinence was the most common reason for presentation [62]. In this series, spina bifida described the vertebral arch defect, whereas spinal dysraphism represented an additional diagnosis based on the clinical signs and morphological spinal cord abnormalities [62]. The phenotype reported by Wilson et al. [62] was anatomically and clinically more extensive than the limited T1 dorsal arch defects reported in Pugs by Ryan et al. [10].

A breed-associated molecular finding has been reported for spinal dysraphism in Weimaraners. Safra et al. [63] performed genome-wide association mapping using four affected and 96 unaffected Weimaraners and identified an approximately 1.5-Mb associated region on canine chromosome 8 containing *NKX2-8*. An *NKX2-8* A150fs frameshift variant segregated with the mapped phenotype in the investigated family material. The affected Weimaraners displayed the characteristic neurological phenotype of spinal dysraphism, and spina bifida was not observed in the Weimaraner cases [63]. One additional Weimaraner with ataxia and paraparesis did not carry the *NKX2-8* A150fs variant; in the absence of diagnostic imaging or histopathological confirmation, Safra et al. [63] considered the diagnosis of spinal dysraphism in this animal suggestive rather than definitive [63].

Published feline evidence for thoracic dorsal arch defects is limited. No spina bifida was identified in the retrospective radiographic series of 200 cats reported by Newitt et al. [54]. Tamura et al. [64] described two young cats with tethered cord syndrome secondary to spina bifida aperta. In case 1, the fistula was connected to the dural sac at the conus medullaris with caudal tethering, and histopathological examination confirmed a meningomyelocele; in case 2, a tubular structure was connected to the dural sac at the thoracic spinal cord with dorsal tethering, and histopathological examination confirmed a meningocele [64]. This two-case report documents two anatomically distinct forms of feline spina bifida aperta with tethered cord syndrome but does not provide prevalence data.

Thoracic dorsal arch abnormalities can be characterized by the affected vertebral level, the width and completeness of the osseous defect, the morphology of the laminae and spinous process, and the presence of fusion with adjacent dorsal processes. Associated abnormalities may involve the skin, subcutaneous tissues, meninges, spinal cord, or other midline structures [10,30,46,62,63,64]. These anatomical features distinguish isolated osseous abnormalities, including bifid spinous processes, from dysraphic malformations with meningeal or neural involvement.

#### 5.3.5. Rib Abnormalities Associated with Thoracic Vertebral Malformations

The ribs are integral components of thoracic regional morphology because they articulate with the thoracic vertebrae and contribute to the structural organization of the thoracic cage [1,2,32,33]. At axial boundaries, atypical costal morphology may be compatible with altered regional identity [7,8,27,28,29]. Costal abnormalities associated with vertebral malformations may include variation in rib number, morphology, attachment, or orientation [1,2,11,59]. At the cervicothoracic and thoracolumbar boundaries, atypical costal morphology may form part of a transitional vertebral configuration rather than an isolated rib abnormality.

Boundary-associated costal abnormalities include cervical ribs arising from C7 and unilateral or bilateral ribs or rib-like structures associated with L1 at the thoracolumbar transition. In these configurations, costal morphology contributes to regional characterization of the corresponding vertebra together with transverse-process morphology, the configuration of the adjacent vertebral regions, and whole-column vertebral enumeration [10,30,45,54].

Thoracolumbar costal variation has been documented in cats. Newitt et al. [54] identified thoracization of L1 in 18 of 200 cats examined radiographically. The additional ribs arising from L1 varied in morphology and were frequently small or fragmented [54]. Their size and irregular appearance could lead to confusion with acquired rib fractures and could also affect vertebral enumeration and anatomical localization [54]. Pollard et al. [45] similarly documented a rib arising from L1 in one of 17 kink-tailed cats in which the complete vertebral column was examined radiographically. In both studies, the rib arising from L1 was described as part of thoracolumbar transitional morphology [45,54].

A less common configuration was reported by Tremolada et al. [59] in a small series of six cats with persistent right aortic arch. Four of the six cats had at least one concurrent axial skeletal malformation. In one animal, 15 ribs were present on the left side of the thorax, with two ribs articulating with the same vertebra [59]. Other cats in the series exhibited altered thoracic or lumbar vertebral number, thoracic vertebral body malformations, caudal vertebral abnormalities, or a supernumerary sternebra [59].

The principal imaging studies of thoracic vertebral malformations in brachycephalic dogs were designed primarily to classify vertebral body formation defects, block vertebrae, transitional vertebrae, and dorsal arch abnormalities [9,10]. Rib abnormalities were not analyzed as an independent systematic outcome in these studies [9,10]; consequently, they do not provide prevalence estimates specifically for congenital rib abnormalities in French Bulldogs, English Bulldogs, or Pugs. The presence of a thoracic vertebral malformation alone does not establish the presence of an associated rib abnormality.

Vertebral angulation or rotation may also alter the spatial orientation of ribs articulating with the affected thoracic segments [1,2,11]. This configuration is morphologically distinct from abnormalities involving rib number, size, continuity, or vertebral attachment. Describing these features separately avoids assigning a developmental origin solely from postnatal imaging. Anatomical characterization can include the number of ribs on each side, their vertebral levels of origin, bilateral symmetry, length, continuity, orientation, and vertebral articulation. The first and last rib-bearing segments are particularly relevant because transitional morphology may alter regional identification [10,30,45,54]. Small or fragmented rib-like structures may also mimic acquired rib injury, as specifically reported for thoracicization of L1 in cats [54].

Congenital costal abnormalities can be described according to rib number, morphology, symmetry, vertebral level of origin, and articulation. At axial boundaries, these features may occur as components of transitional morphology compatible with altered regional identity [7,8,27,28,29,30,45,54]. Current canine and feline evidence provides limited information on the independent clinical significance and genetic determinants of congenital rib abnormalities.

#### 5.3.6. Thoracic Caudal Articular Process Dysplasia and Its Relationship to Pug-Associated Thoracolumbar Myelopathy

In the studies considered here, caudal articular process dysplasia was characterized as hypoplasia or aplasia of one or more caudal vertebral articular processes [11,65]. The abnormality may be unilateral or bilateral and may affect an isolated vertebra or multiple thoracic vertebrae [65]. Because each caudal articular process normally articulates with the cranial articular process of the immediately caudal vertebra, hypoplasia or aplasia results in incomplete or absent formation of the corresponding zygapophyseal articulation [1,2,11,65]. Computed tomography permits morphological characterization of caudal articular process hypoplasia or aplasia and assessment of its anatomical distribution [11,65].

Thoracic caudal articular process dysplasia is common in neurologically normal brachycephalic dogs. In the retrospective computed tomographic study by Bertram et al. [65], the abnormality was identified in 76 of 108 French Bulldogs (70.4%), 53 of 63 English Bulldogs (84.1%), and 97 of 100 Pugs (97.0%). Compared with the two Bulldog populations, Pugs had a higher frequency of complete aplasia, a greater number of affected vertebrae per dog, and more frequent bilateral and generalized involvement [65]. The anatomical distribution also differed among breeds, with Pugs showing a marked concentration of caudal articular process aplasia between T10 and T13, a region that was largely spared in the French and English Bulldogs examined [65].

The occurrence of caudal articular process dysplasia in 97.0% of neurologically normal Pugs demonstrates that this osseous phenotype can be present without clinically evident thoracolumbar spinal cord disease [65]. Chronic low-grade vertebral instability associated with caudal articular process dysplasia has been proposed as a potential mechanism contributing to thoracolumbar spinal disease in Pugs [66,67,68]. However, the cited studies did not directly demonstrate abnormal vertebral motion, and both Driver et al. [67] and Nishida et al. [68] emphasized that a causal relationship between caudal articular process dysplasia and the associated spinal disorders had not been established.

Fisher et al. [66] retrospectively described 11 Pugs with neurological dysfunction associated with hypoplastic or aplastic thoracolumbar caudal articular processes and constrictive fibrous tissue surrounding the spinal cord, together with five Pugs that had comparable articular process abnormalities without neurological dysfunction. The authors proposed that chronic abnormal segmental motion associated with the articular process malformations contributed to the development of constrictive fibrosis [66]. The proposed instability–fibrosis relationship remained speculative in this retrospective series, particularly because comparable caudal articular process abnormalities were also identified in five Pugs without neurological dysfunction [66].

Driver et al. [67] subsequently evaluated 18 Pugs with chronic progressive thoracolumbar myelopathy and computed tomographic confirmation of caudal articular process dysplasia. At the intervertebral level corresponding to the principal spinal cord lesion, 17 dogs had bilateral caudal articular process aplasia and one had unilateral aplasia with severe contralateral hypoplasia [67]. Spinal cord compression was identified in 17 dogs and was associated with intervertebral disc protrusion in ten, a suspected spinal arachnoid diverticulum in four, and suspected pia-arachnoid fibrosis in three [67]. In 16 of 18 dogs (89%), caudal articular process dysplasia was also present at one or more additional vertebral levels without corresponding magnetic resonance imaging (MRI) abnormalities [67]. Although bilateral caudal articular process dysplasia was present at the level of the principal MRI abnormality in all 18 dogs, Driver et al. [67] concluded that a causal relationship between caudal articular process dysplasia and the causes of thoracolumbar myelopathy was not confirmed by their study.

The relationship with intervertebral disc herniation was investigated by Nishida et al. [68] in 57 Pugs. Caudal articular process hypoplasia or aplasia was identified in 52 dogs (91.2%), with the abnormalities concentrated predominantly between T10 and T13. Colocalization with thoracolumbar intervertebral disc herniation occurred in 11 dogs (19.3%). At the intervertebral-space level, disc herniation was identified at 12.3% of spaces associated with abnormal caudal articular processes compared with 2.4% of spaces associated with normal processes [68]. These findings demonstrate a statistical association within the examined cohort but do not establish that caudal articular process dysplasia causes disc degeneration or herniation; the authors themselves emphasized that a larger cohort would be required to test causality.

The pathological heterogeneity of Pug-associated thoracolumbar myelopathy was demonstrated prospectively by Wachowiak et al. [69]. Thirty-two Pugs with chronic progressive T3–L3 myelopathy underwent computed tomography and magnetic resonance imaging, and postmortem examination was available in a subset [69]. All 32 dogs had caudal articular process dysplasia at more than one vertebral level within the T3–L3 region [69]. MRI identified constrictive myelopathy alone in three dogs, constrictive myelopathy together with at least one additional myelopathy in 17, non-constrictive myelopathies without constrictive myelopathy in 11, and no detectable spinal cord lesion in one [69]. Nineteen of the 32 dogs had more than one type of myelopathy. No apparent relationship was identified between the anatomical distribution of caudal articular process dysplasia and either the location of the most severe myelopathy or the type of spinal cord disease [69].

Collectively, these studies document a heterogeneous spectrum of thoracolumbar spinal disease in Pugs with caudal articular process dysplasia. Reported abnormalities include constrictive myelopathy with meningeal fibrosis, intervertebral disc protrusion or herniation, spinal arachnoid diverticula, and focal or multifocal spinal cord compression, occurring alone or in combination [66,67,68,69]. Caudal articular process dysplasia was colocalized with spinal cord lesions in some affected cohorts but was also present at additional vertebral levels without corresponding lesions and in neurologically normal Pugs [65,67,69]. Its specific contribution to individual thoracolumbar myelopathies remains unresolved.

A genetic contribution to the broader Pug thoracolumbar myelopathy phenotype was investigated by Brander et al. [70] in a cohort comprising 51 affected and 38 control Pugs. Using a Bayesian complex-trait mapping approach, the authors identified 19 associated loci containing 67 genes, including 34 potential candidate genes, while an independent selection-signature analysis identified three candidate regions under selection [70]. Candidate genes within these regions were associated with biological functions relevant to bone homeostasis, cartilage biology, fibrosis, and inflammatory responses [70]. These findings support a complex genetic contribution to Pug-associated thoracolumbar myelopathy and are consistent with the multifactorial nature of the phenotype investigated. The number of retained loci depended on the effect-size threshold applied: 19 loci were identified from the 50 variants with the largest estimated effects, whereas restriction to the ten largest-effect variants would have retained seven loci [70]. The study identified associated loci and candidate genes for the broader clinical Pug thoracolumbar myelopathy phenotype rather than causal variants for a specific anatomical lesion [70]. The results therefore provide evidence of complex genetic susceptibility to Pug-associated thoracolumbar myelopathy but do not establish whether caudal articular process dysplasia, meningeal fibrosis, and other constituent abnormalities share the same genetic determinants.

Thoracic caudal articular process dysplasia can be characterized by the affected vertebral levels, laterality, distinction between hypoplasia and aplasia, number of involved segments, and morphology of the corresponding facet articulations. In neurologically affected dogs, associated intervertebral disc, meningeal, vertebral canal, and spinal cord abnormalities provide separate components of the anatomical phenotype [65,66,67,68,69]. This distinction separates a common congenital osseous abnormality from the heterogeneous spinal cord disorders reported in Pugs with chronic thoracolumbar myelopathy. No analogous feline breed-associated syndrome was identified in the literature reviewed.

### 5.4. Lumbar and Lumbosacral Vertebral Anomalies

The lumbar region normally comprises seven vertebrae in dogs and cats and forms the mobile presacral portion of the vertebral column between the rib-bearing thoracic region and the sacrum [1,2,32,33]. Congenital abnormalities affecting this region include variation in lumbar vertebral number, transitional morphology at the lumbosacral boundary, vertebral body formation defects, congenital vertebral fusion, and dorsal arch or process abnormalities [3,54]. Accurate regional classification depends on the complete presacral vertebral formula because an apparently atypical terminal lumbar vertebra may represent numerical variation, transitional morphology at the lumbosacral boundary, or part of a broader axial pattern involving another vertebral region [36,37,54].

The lumbosacral junction has a distinctive structural role because it connects the mobile lumbar column with the sacrum and pelvis and contributes to transmission of forces between the vertebral column and pelvic limbs [1,2,35,36,37]. Morphological asymmetry at this boundary may be associated with altered relationships among the terminal presacral vertebra, sacral wings, ilium, and sacroiliac articulations [35,36,37]. Nevertheless, congenital morphology does not by itself establish pathological significance. Associated pathological findings may include intervertebral disc degeneration, spondylosis, vertebral canal or foraminal stenosis, and compression of the cauda equina, and can be described separately from the congenital vertebral configuration [36,37,71].

#### 5.4.1. Lumbosacral Transitional Vertebrae

A lumbosacral transitional vertebra (LTV) is a congenital vertebral segment situated at the boundary between the last morphologically typical lumbar vertebra and the sacrum and exhibiting a combination of lumbar and sacral characteristics [36,37,71]. The abnormality may be symmetrical or asymmetrical and may involve the transverse processes or sacral wings, spinous processes, sacroiliac attachment, and fusion between sacral components [36,37,72]. The terms sacralization of the terminal lumbar vertebra and lumbarization of the first sacral segment are frequently used descriptively.

Definitions and imaging criteria for lumbosacral transitional vertebrae vary among studies. Damur-Djuric et al. [36] classified LTV primarily according to the morphology and symmetry of the transverse processes and their relationship with the ilium, whereas the expanded classification used by Lappalainen et al. [72] additionally included separation of the S1 spinous process from the median sacral crest and a short, caudally positioned eighth lumbar vertebra within the LTV complex. Reported frequencies consequently depend on the classification system, imaging projections, anatomical structures evaluated, and study population.

Damur-Djuric et al. [36] classified the transverse processes of canine lumbosacral transitional vertebrae as lumbar, intermediate, or sacral type. The lumbar type retains predominantly lumbar transverse-process morphology, the intermediate type shows characteristics between those of a lumbar transverse process and a sacral wing, and the sacral type resembles a sacral wing and is closely related to the ilium. A transitional vertebra is classified as symmetrical when both transverse processes have the same morphology and as asymmetrical when the right and left processes belong to different categories [36]. Highly asymmetrical transitional vertebrae may also be angulated relative to the adjacent vertebral segments [36].

Radiographic projection and superimposition of the iliac wings may affect the apparent morphology and visibility of the transverse processes. Classification based exclusively on a ventrodorsal pelvic radiograph may therefore incompletely represent three-dimensional osseous anatomy [72]. In the computed tomographic subset examined by Lappalainen et al. [72], radiographic classification would have changed in two dogs, while a small unilateral transverse process not visible radiographically was identified in another. These observations demonstrate the additional anatomical information provided by multiplanar imaging when radiographic superimposition limits characterization of the lumbosacral junction [72].

In a retrospective evaluation of pelvic radiographs from 4000 medium- and large-breed dogs representing 144 breeds and submitted for canine hip-dysplasia screening, Damur-Djuric et al. [36] identified lumbosacral transitional vertebrae in 138 dogs (3.5%). Symmetrical and asymmetrical forms occurred with similar frequency, and no sex predisposition was detected. German Shepherd Dogs and Greater Swiss Mountain Dogs were more frequently affected than the remaining breed population [36]. The overall 3.5% frequency therefore describes this population of medium- and large-breed dogs submitted for hip-dysplasia screening rather than an unselected canine population.

The developmental interpretation of lumbosacral transitional morphology remains incompletely resolved. Damur-Djuric et al. [36] proposed that an LTV may represent an autonomous intermediate vertebral type rather than a simple transformation of an otherwise typical lumbar vertebra into a sacral vertebra, or vice versa. They further hypothesized that variation in the position at which the developing pelvis contacts the vertebral column could influence the morphology of the developing vertebral segment [36]. This interpretation provides a plausible developmental framework for LTV morphology but remains hypothetical, as it was inferred from postnatal radiographic findings rather than demonstrated through embryological or molecular evidence.

Variation in lumbar vertebral number may form part of the lumbosacral transitional phenotype depending on the classification system used. Lappalainen et al. [72] evaluated ventrodorsal pelvic and laterolateral lumbar radiographs from 228 German Shepherd Dogs and classified 92 dogs (40.4%) as having a lumbosacral transitional abnormality under their expanded classification. Ten dogs had eight lumbar vertebrae. In six of these dogs, a short and caudally positioned L8 was the only abnormal radiographic finding, whereas four also had incomplete fusion between S2 and S3 [72]. One additional dog had six lumbar vertebrae. The authors considered a short and caudally positioned L8 to be part of the lumbosacral transitional complex rather than an isolated numerical variant.

The comparatively high frequency reported by Lappalainen et al. [72] was strongly influenced by the phenotype definition and imaging protocol. Separation of the S1 spinous process from the median sacral crest was included as a transitional abnormality and accounted for 62 of the 92 affected dogs. All dogs with eight lumbar vertebrae were identified only on the laterolateral projection, and combining laterolateral with ventrodorsal radiography significantly improved diagnostic sensitivity compared with the ventrodorsal projection alone [72]. These findings demonstrate that frequencies reported for lumbosacral transitional vertebrae cannot be compared directly without considering classification criteria and imaging methodology.

A lumbosacral transitional vertebra does not inevitably result in clinically significant lumbosacral disease. Nevertheless, Flückiger et al. [71] demonstrated an epidemiological association with cauda equina syndrome. Among 4000 control dogs without signs of cauda equina syndrome, 138 (3.5%) had an LTV, compared with 15 of 92 dogs (16.3%) affected by the syndrome. Dogs with an LTV were reported to be approximately eight times more likely to have cauda equina syndrome than dogs without an LTV, and in affected dogs carrying an LTV the lesion responsible for the syndrome was located between the last morphologically typical lumbar vertebra and the transitional segment [71]. Dogs with an LTV also developed the syndrome approximately one to two years earlier than affected dogs without an LTV.

These findings support a predisposing association rather than deterministic causation. Altered biomechanics at the functional lumbosacral junction have been proposed as a possible explanation for this association [71,72], although these studies did not directly quantify abnormal segmental motion or load transmission. In clinically affected animals, LTV may coexist with intervertebral disc degeneration, vertebral canal or foraminal abnormalities, and neural compression; these associated pathological findings can be characterized separately from the congenital transitional morphology [71,72].

Lumbosacral transitional vertebrae have also been documented in cats. Newitt et al. [37] retrospectively identified 14 cats with transitional vertebrae and compared them with 100 cats without this abnormality. Six of the 14 affected cats showed asymmetry or rotation of the sacroiliac attachment in the dorsal plane; four of these six had lumbosacral spondylosis and two also had hip dysplasia [37]. No additional degenerative abnormalities were identified in the remaining eight cats with transitional vertebrae. Lumbosacral spondylosis was more frequent among cats with transitional morphology, particularly those with pelvic asymmetry or rotation, but this difference was not statistically significant. Hip dysplasia occurred with a similar frequency in cats with and without transitional vertebrae [37].

Harris et al. [73] subsequently compared 13 cats with lumbosacral vertebral canal stenosis with 405 cats undergoing computed tomography for reasons unrelated to spinal disease. Lumbosacral transitional vertebrae were present in seven of the 13 cats with stenosis (53.8%) and in 24 of the 405 controls (5.9%). Transitional morphology was significantly associated with lumbosacral stenosis (*p* < 0.0001; odds ratio 18.52; 95% confidence interval 6.1–62.1) [73]. No significant influence of breed or sex was identified, and the difference in age at diagnosis between affected cats with and without an LTV was not statistically significant [73]. Harris et al. [73] concluded that LTV could be considered a risk factor for the development of lumbosacral vertebral canal stenosis in cats.

Genetic studies support an inherited contribution to canine lumbosacral transitional morphology, although heritability estimates vary among populations and analytical approaches. Gluding et al. [74] analyzed radiographic screening data from 27,579 German Shepherd Dogs and estimated a moderate heritability of h^2^ = 0.27 for the comprehensive LTV phenotype. Estimates for individual morphological categories were lower, while positive additive genetic correlations were identified among several LTV categories [74]. These findings support inherited susceptibility within the investigated German Shepherd Dog population but do not identify a specific causal gene.

More recently, Berg et al. [75] estimated pedigree-based heritability for LTV in nine dog breeds and obtained breed-specific values ranging from 0.056 to 0.314. The lowest estimate occurred in Norwegian German Shepherd Dogs and the highest in Brittany dogs, while genetic correlations between LTV and canine hip dysplasia varied among breeds and were predominantly non-significant [75]. The German Shepherd Dog estimates differed markedly between Gluding et al. [74] and Berg et al. [75], which examined different populations and used different datasets and analytical frameworks.

Lumbosacral transitional vertebrae can be characterized by total lumbar vertebral count, morphology of the terminal presacral vertebra, symmetry and configuration of the transverse processes or sacral wings, relationship with the ilium, fusion of sacral components, and alignment of the sacroiliac articulations [36,37,71,72]. The imaging projections and classification criteria used are relevant because diagnostic sensitivity and morphological categorization vary with the imaging protocol [72]. Computed tomography can provide additional characterization of osseous morphology when radiographic superimposition limits assessment [72]. In clinically affected animals, the functional lumbosacral intervertebral space and associated disc degeneration, spondylosis, vertebral canal or foraminal stenosis, and cauda equina compression represent separate pathological findings [36,37,71,73]. This component-based description separates congenital transitional morphology from associated degenerative or compressive abnormalities.

#### 5.4.2. Lumbar Vertebral Number and Vertebral Body Formation Defects

Variation in lumbar vertebral number and abnormalities of vertebral body formation represent anatomically distinct congenital configurations. Numerical variation changes the number of vertebrae assigned morphologically to the lumbar region, whereas a formation defect alters the morphology of an individual vertebral body. The two configurations may coexist, and an atypical terminal presacral segment alone does not establish either numerical variation or an intrinsic vertebral body formation defect. Regional classification depends on enumeration of the presacral vertebral column and assessment of both the thoracolumbar and lumbosacral boundaries because a count of six or eight morphologically lumbar vertebrae may occur together with transitional morphology at an adjacent axial boundary [1,2,32,33,50,72,76].

Lumbar numerical variation has been documented in dogs. In the radiographic study of 73 French Bulldogs by Kuricová et al. [50], five dogs had eight vertebrae classified as lumbar and two had six. Numerical variation was also identified in the thoracic region of the same population, reinforcing the need to interpret an atypical lumbar count within the complete presacral vertebral formula rather than as an isolated regional observation. In German Shepherd Dogs, Lappalainen et al. [72] identified eight lumbar vertebrae in ten of 228 dogs and six lumbar vertebrae in one. However, the authors included a short and caudally positioned L8 within an expanded classification of lumbosacral transitional abnormalities. These findings illustrate that an identical regional count may be classified differently depending on the morphology of the terminal vertebra, the phenotype definition, and the imaging projections used.

Feline lumbar numerical variation was evaluated by Thanaboonnipat et al. [76] in a retrospective review of abdominal radiographs from 1365 cats. Congenital lumbosacral abnormalities were identified in 226 cats; 207 had a single recorded congenital abnormality and 19 had multiple abnormalities. Among cats with a single congenital abnormality, six lumbar vertebrae were recorded in 141 animals and five lumbar vertebrae in one. Other configurations included sacralization in 48 cats, lumbarization in 16, and a hemivertebra in one [76]. Because the study was based on abdominal radiographs rather than systematic whole-column imaging, the reported categories primarily describe regional radiographic configurations [76]. In this context, an altered lumbar count can be interpreted only in relation to the morphology of the thoracolumbar and lumbosacral boundaries.

Thanaboonnipat et al. [76] also reported a statistical association between congenital lumbosacral abnormalities, considered collectively, and radiographic large-bowel abnormalities (*p* = 0.0069; odds ratio = 1.920). The congenital category included numerical variation, sacralization, lumbarization, multiple concurrent abnormalities, and the single hemivertebral case. The reported association therefore applies to this composite category rather than to a specific morphological subtype. In particular, the contribution of six lumbar vertebrae or any other individual configuration to the gastrointestinal finding cannot be evaluated independently from these data. The retrospective association reported in this study does not establish a direct causal relationship between congenital lumbosacral morphology and constipation or megacolon [76].

Lumbar vertebral body formation defects may involve partial absence or hypoplasia of ventral, lateral, or combined regions of the vertebral body. As in the thoracic region, these abnormalities can be described according to the location, direction, and extent of deficient vertebral tissue rather than solely by the variably applied term hemivertebra [3,9,11]. Depending on the direction and severity of the defect, the affected vertebra may be wedge-shaped or asymmetrical and may be associated with focal kyphotic, lordotic, scoliotic, or combined angular deformation. Postnatal imaging can characterize the morphology and structural consequences of the defect but does not, by itself, establish the precise embryological or molecular mechanism responsible for an individual malformation.

Published canine lumbar vertebral body formation defects are represented predominantly by individual clinical reports rather than population-based studies. Musser et al. [77] described a German Shepherd Dog with two abnormalities classified by the authors as dorsal hemivertebrae, located at T10 and L3. The L3 malformation was associated with moderate focal kyphosis and scoliosis, and the vertebral canal at the adjacent L2–L3 and L3–L4 intervertebral spaces was approximately 70% of its normal diameter [77]. MRI additionally demonstrated marked dorsal subarachnoid-space dilatation at L3 and intramedullary T2-weighted hyperintensity at L1 [77]. The concurrent T10 hemivertebra was also associated with marked angular deformation and spinal cord compression [77]. This case illustrates that a pronounced lumbar vertebral body formation defect may coexist with angular deformation, reduced vertebral canal dimensions, and spinal cord abnormalities [77]. As a single clinical case involving two concurrent vertebral malformations, the report documents the structural and neurological expression of these abnormalities but provides no estimate of their population frequency [77].

Published feline evidence is even more limited. Thanaboonnipat et al. [76] recorded a single hemivertebra among the 1365 cats examined. The available report did not further characterize its vertebral level, directional subtype, relationship with spinal alignment, or associated pathological significance. This finding documents the occurrence of a vertebral body formation defect within the examined feline population, while the anatomical characteristics and frequency of specifically lumbar hemivertebral phenotypes remain insufficiently characterized.

Lumbar numerical variation can be characterized according to the presacral vertebral formula, morphology of the thoracolumbar and lumbosacral boundary segments, and method used for vertebral enumeration. Identification of both the thoracolumbar and lumbosacral boundaries provides the anatomical context required for regional vertebral enumeration, particularly when transitional morphology is present [50,72,76]. Vertebral body formation defects can be described separately according to the affected level, deficient portion of the vertebral body, distinction between aplasia and hypoplasia, symmetry, and associated spinal curvature.

Computed tomography can provide detailed characterization of osseous morphology, vertebral canal dimensions, and complex three-dimensional deformation [72,77], whereas magnetic resonance imaging can demonstrate associated spinal cord, subarachnoid, and intramedullary abnormalities [77]. This component-based approach distinguishes regional numerical variation from vertebral body formation defects and describes associated structural or neurological abnormalities separately from the congenital osseous morphology.

#### 5.4.3. Lumbar Dorsal Arch Defects and Lumbosacral Spinal Dysraphism

Lumbar dorsal arch defects are characterized by incomplete dorsal midline formation or union of the vertebral laminae and may be expressed as a limited osseous cleft, a bifid spinous process, or a broader defect involving the dorsal boundary of the vertebral canal [3,46,62]. As in other vertebral regions, the presence of an osseous defect alone does not establish involvement of the spinal cord or meninges [46,62]. Lumbar spina bifida may occur as an isolated osseous vertebral arch abnormality or as part of a more extensive dysraphic malformation involving meningeal or neural structures [46,62].

Spinal dysraphism encompasses a broader spectrum of congenital abnormalities involving the spinal cord, meninges, vertebral arches, and associated dorsal midline tissues [46,62]. In closed forms, the dysraphic abnormality remains covered by skin, although abnormalities involving the spinal cord, meninges, tethering, or associated dorsal midline tissues may still be present [46]. Open forms involve incomplete cutaneous coverage or direct exposure of dysraphic tissues [46]. Spina bifida occulta denotes incomplete vertebral arch closure without protrusion of neural tissue, whereas closed spinal dysraphism encompasses a broader range of skin-covered abnormalities that may include neural or meningeal involvement [46]. Conversely, spina bifida aperta denotes an open defect in which meningeal or neural structures may be exposed or protrude through the osseous abnormality [46,62,78,79,80].

Protrusion of the meninges through a vertebral defect is termed a meningocele, whereas a protruding sac containing meninges together with neural tissue is described as a meningomyelocele or myelomeningocele [62,78,79,80]. Because terminology varies among publications, specification of the anatomical contents of the protruding structure provides a more precise morphological description when these have been established by imaging, surgery, or histopathological examination.

Lumbosacral meningocele and meningomyelocele have been documented in Bulldogs. Martín Muñiz et al. [78] retrospectively described six affected dogs, comprising five French Bulldogs and one English Bulldog. Magnetic resonance imaging identified a meningocele in two dogs and a meningomyelocele in four. All six dogs had a visible lumbosacral dimple and urinary and fecal incontinence, while three also had mild or moderate paraparesis [78]. This six-dog case series documents lumbosacral meningocele and meningomyelocele in French and English Bulldogs but does not provide an estimate of prevalence or breed-specific risk [78].

All six dogs underwent surgical treatment of the dysraphic defect [78]. Urinary and fecal continence improved in two dogs and remained unchanged in four, while paraparesis improved in two of the three dogs in which it had been present. These findings illustrate variable postoperative neurological outcomes in this surgically treated series. The study was a surgical case series and did not include a conservatively managed comparison group [78].

A severe open lumbar dysraphic phenotype was characterized by Chen et al. [79] in a Poodle fetus identified during a twin pregnancy. After birth, the affected puppy had paraplegia and a linear dorsal midline defect extending from L2 to L6, associated with open spina bifida and myelomeningocele [79]. Imaging and postmortem examination demonstrated additional congenital abnormalities involving the thoracic and lumbar vertebrae, sacrum, scapula, and internal organs [79]. This case illustrates the potential anatomical extent of severe open spinal dysraphism and its association with multisystem congenital abnormalities.

Lumbar spina bifida with neural involvement has also been documented in cats. Muller et al. [80] described a 2.5-year-old Domestic Shorthair cat with chronic progressive paraparesis and urinary incontinence associated with an L5 dorsal midline lesion. Magnetic resonance imaging demonstrated a tract extending from the epidermis through a split L5 spinous process to the dorsal meninges, with dorsal displacement and tethering of the spinal cord. Marked syringohydromyelia was present at L4, together with mild intramedullary contrast enhancement at L5 interpreted as consistent with myelitis [80]. The imaging findings were considered consistent with spina bifida at L5 associated with a meningomyelocele and tethered cord.

Surgical excision and decompression were performed, and histopathological examination demonstrated fibrocartilaginous connective tissue containing peripheral nerves and vascular structures, confirming the diagnosis of meningomyelocele [80]. Six months after surgery, the cat remained non-ambulatory paraparetic and urinary incontinent but showed improved comfort and mobility using the thoracic limbs. This single case documents complex neural and meningeal involvement associated with feline lumbar spina bifida, including meningomyelocele, tethered cord, and syringohydromyelia [80].

The pathological significance of a lumbar dorsal arch defect therefore depends primarily on its anatomical relationships and associated tissues rather than on the osseous cleft alone. A limited skin-covered osseous defect may occur without demonstrated neural involvement, whereas dysraphic abnormalities involving a dermal tract, meningocele, meningomyelocele, tethered spinal cord, syringohydromyelia, myelitis, or intrinsic spinal cord abnormalities may be associated with neurological dysfunction [62,78,79,80]. The external appearance or apparent osseous severity of the defect alone does not reliably define the extent of neural involvement. Radiography may demonstrate abnormalities of the vertebral arches or spinous processes, whereas magnetic resonance imaging can define involvement of the meninges and spinal cord, identify dysraphic tracts or tethering, and demonstrate associated intramedullary abnormalities [78,79,80]. Histopathological examination may provide definitive characterization of the tissues contained within a dysraphic protrusion when tissue is available.

Lumbar dorsal arch abnormalities can be characterized by the affected vertebral levels, width and completeness of the osseous defect, morphology of the laminae and spinous processes, integrity of the overlying skin and subcutaneous tissues, and the presence of a dorsal midline dimple, mass, sinus, or fistulous tract. Meningeal or neural protrusion, spinal cord tethering, and associated intramedullary abnormalities provide additional components of the dysraphic phenotype [62,78,79,80]. This component-based description distinguishes an isolated osseous dorsal arch abnormality from a broader spinal dysraphism involving meningeal or neural structures.

#### 5.4.4. Lumbar Block Vertebrae and Congenital Segmental Fusion

Block vertebrae are congenital malformations characterized by partial or complete fusion of two or more adjacent vertebral segments [3,11,60,61]. Block formation involves loss of segmentation between adjacent vertebral segments and may affect the vertebral bodies with variable involvement of the vertebral arches and other vertebral components [3,11,61]. From a developmental perspective, this morphology is compatible with disturbed segmentation or separation of adjacent vertebral primordia. Because abnormalities of vertebral formation and segmentation may coexist, fused segments may also exhibit hypoplasia, asymmetry, or other intrinsic morphological defects [3,11,61].

Congenital block formation is morphologically distinct from acquired osseous bridging between previously separate vertebral segments [3,11]. Congenital segmentation defects show osseous continuity between adjacent vertebral components with partial or complete loss of the expected intervertebral separation, whereas acquired bridging develops between previously distinct segments in association with degenerative, inflammatory, traumatic, or postoperative changes [3,11]. Morphologically asymmetric congenital fusion may be associated with angular deformation, but postnatal imaging alone does not establish the developmental sequence by which that deformation arose.

Published lumbar-specific evidence is limited. Available canine reports include complex thoracolumbar formation–segmentation defects involving L1 and an individual case of lumbar block vertebrae [61,81], whereas the feline reports cited in this review document block vertebrae without establishing a specifically lumbar phenotype [54,60]. The available lumbar-specific evidence therefore consists predominantly of individual clinical observations or complex malformations involving the thoracolumbar boundary [61,81]. This evidence base does not permit estimation of lumbar-specific prevalence, breed distribution, or typical clinical significance.

Couto et al. [61] described a complex congenital malformation involving T13–L1 in an adult Rottweiler with progressive thoracolumbar myelopathy. The T13 and L1 vertebral bodies were abnormally formed and non-segmented, whereas the corresponding vertebral arches remained separate. The abnormality was associated with marked focal kyphosis, vertebral canal stenosis, and ventral spinal cord compression, and adjacent intervertebral disc degeneration and vertebral endplate sclerosis were also present cranial to the malformation [61]. The lesion represented a combined defect of vertebral body formation and segmentation associated with marked angular deformation, vertebral canal stenosis, and spinal cord compression [61]. This case formed part of a three-dog retrospective series selected for neurologically significant combined formation and segmentation defects [61]. The study documented adjacent intervertebral disc degeneration but did not directly test a mechanical causal relationship with the congenital malformation [61].

A specifically lumbar block-vertebra phenotype was reported by Sundaram et al. [81] in an 18-month-old female Cocker Spaniel with multiple congenital abnormalities. Contrast radiography demonstrated lumbar scoliosis attributed by the authors to block vertebrae, together with only two hypoplastic caudal vertebrae. The dog also had anury, type II atresia ani, a rectovestibular fistula, and vulvar hypoplasia, but no detectable neurological deficits [81]. The authors interpreted this constellation of abnormalities within the broader context of caudal regression syndrome. The reported phenotype combined lumbar block vertebrae and scoliosis with marked reduction of the caudal vertebral column and anorectal and urogenital abnormalities [81]. Despite these abnormalities, no neurological deficits were detected [81].

Block vertebrae have also been documented in cats. Newitt et al. [54] identified block vertebrae in three cats within a radiographic population of 200 animals, whereas Crowe et al. [60] described multiple thoracic block vertebrae in a young cat with lordosis and clinically significant T3–T4 intervertebral disc protrusion. In the latter case, the authors considered angular deformation and adjacent-segment disease likely contributors to the disc degeneration and protrusion but explicitly acknowledged the limitations of causal inference from a single animal with an unknown previous history [60]. The fused segments reported by Crowe et al. [60] were exclusively thoracic and therefore do not document a lumbar feline block-vertebra phenotype.

Clinical expression varies among the reported block-vertebra phenotypes. In the feline series cited by Crowe et al. [60], the three cats with block vertebrae reported by Newitt et al. [54] had no associated clinical signs despite marked scoliosis, and the Cocker Spaniel described by Sundaram et al. [81] had lumbar scoliosis without detectable neurological deficits. By contrast, the complex T13–L1 formation–segmentation defect reported by Couto et al. [61] was associated with severe kyphosis, vertebral canal stenosis, and spinal cord compression. Adjacent intervertebral disc degeneration was reported in the cases of Crowe et al. [60] and Couto et al. [61], but these observations do not establish a direct biomechanical causal relationship.

Anatomical characterization of lumbar block vertebrae includes the affected vertebral levels, number of fused segments, completeness and symmetry of fusion, vertebral components involved, and the presence or absence of a residual intervertebral boundary or disc space [3,11,60,61]. Additional findings may include angular deformation, vertebral canal or foraminal narrowing, adjacent intervertebral disc or endplate abnormalities, and concurrent congenital abnormalities involving the sacrum, pelvis, caudal vertebrae, spinal cord, anorectal region, or urogenital structures [60,61,81]. These features distinguish isolated congenital segmentation abnormalities from complex caudal developmental phenotypes and allow associated structural or neurological findings to be described separately from the vertebral fusion.

### 5.5. Sacral and Caudal Vertebral Anomalies

The sacral and caudal regions form the terminal portion of the vertebral column but have distinct anatomical and functional relationships. The sacrum provides the principal osseous connection between the vertebral column and pelvis, contributes to the boundaries of the pelvic canal, contains the sacral canal and associated nerve roots, and participates in force transmission between the axial skeleton and pelvic limbs [1,2,32,33]. The caudal vertebrae progressively diminish in size and structural complexity and form the osseous basis of the tail [1,2,32,33].

Congenital abnormalities affecting these regions include partial or complete sacral agenesis, sacrocaudal dysgenesis, dorsal arch abnormalities, abnormal sacral segmentation or fusion, variation in caudal vertebral number, caudal vertebral body formation defects, block vertebrae, and complex caudal developmental phenotypes [37,46,62,81,82,83,84,85]. Because the terminology applied to caudal vertebral malformations varies among studies, precise description of the affected vertebral component is preferable whenever morphology can be defined anatomically.

The biological and pathological significance of these abnormalities depends not only on the number and morphology of the affected vertebrae but also on their relationships with the terminal spinal cord, cauda equina, sacral nerve roots, meninges, sacroiliac articulations, pelvic structures, anorectal and urogenital systems, and associated soft tissues [62,78,79,80,81,82,83,85]. A short or absent tail may represent a predominantly caudal skeletal phenotype, whereas a superficially similar external appearance may accompany extensive sacral, neural, pelvic, or visceral malformation [81,82,83,84,85]. Previous trauma or surgical amputation may produce tail shortening or deformation that resembles a congenital phenotype. Anatomical characterization together with clinical history allows congenital vertebral morphology to be distinguished from acquired tail loss or deformation.

#### 5.5.1. Sacral Agenesis, Sacrocaudal Dysgenesis, and Caudal Regression Phenotypes

Sacral agenesis denotes partial or complete congenital absence of the sacral bones [82]. Sacrocaudal dysgenesis is a broader descriptive term encompassing abnormal development of the sacrum and caudal vertebrae and, in some animals, associated abnormalities of the spinal cord, meninges, and dorsal midline tissues [82,83]. Sacral agenesis and sacrocaudal dysgenesis are anatomically distinct from a lumbosacral transitional vertebra, in which a vertebral segment is present but exhibits mixed lumbar and sacral morphology [36,37,71,72,73,74,75]. Transitional morphology and partial sacral agenesis may nevertheless coexist in the same animal [82].

The term caudal regression syndrome has been used in veterinary literature for complex caudal developmental phenotypes involving variable vertebral, anorectal, urogenital, and appendicular abnormalities [81,82]. Related but anatomically more extensive conditions, such as perosomus elumbis, illustrate the broader spectrum of caudal developmental disruption reported in domestic animals [84]. Because terminology derived from human medicine has been applied variably to veterinary cases, detailed anatomical description provides a more direct characterization of the phenotype. The absent or malformed vertebral levels and associated neural, appendicular, pelvic, and visceral abnormalities provide the principal anatomical components of these complex phenotypes [81,82,84].

Dell’Apa et al. [82] described partial sacral agenesis in two three-month-old female Boxer dogs from the same litter. Both dogs had urinary and fecal incontinence from birth, a short tail stump, an atonic anal sphincter, and absent perineal reflex and sensation, but neither had pelvic-limb paresis or evidence of sciatic nerve dysfunction [82]. The distribution of the neurological abnormalities was consistent with dysfunction involving the sacral spinal cord segments, sacral nerve roots, or cauda equina.

Radiographic examination revealed six morphologically typical lumbar vertebrae followed by a lumbosacral transitional vertebra and a severely hypoplastic vertebra bearing two small sacral transverse processes as the principal sacral remnant [82]. The vestigial sacral segment had an incomplete dorsal arch and lacked a spinous process, while caudal vertebral development was also markedly reduced: cross-sectional imaging identified a single non-articulating coccygeal vertebra in one dog and several non-articulating caudal vertebrae in the other [82]. The phenotype therefore comprised altered morphology at the lumbosacral boundary, severe sacral hypoplasia, dorsal arch non-union, and variable caudal vertebral development. Ultrasonography, computed tomography, and magnetic resonance imaging additionally demonstrated abnormalities of the terminal dural structures, with caudal extension of the dural sac into the subfascial adipose tissue in one dog and an extracanalar subfascial cystic structure communicating with the subarachnoid space and interpreted as a meningocele in the other [82]. Electromyography showed pathological spontaneous activity in the perineal musculature, interpreted as possibly reflecting chronic denervation, whereas the examined pelvic-limb muscles showed no evidence of sciatic nerve involvement [82]. Urinary and fecal incontinence persisted in both dogs, although they otherwise remained clinically stable during approximately three years of follow-up [82]. The occurrence of closely similar abnormalities in two littermates suggested a possible inherited contribution, prompting whole-genome sequencing of one affected dog at approximately 20-fold coverage against 924 control genomes; no strong plausible candidate variant was identified [82]. The genetic basis therefore remains unresolved, with inherited, non-genetic, or combined influences remaining possible.

Sacrocaudal dysgenesis is well documented as a breed-associated phenotype in Manx cats [83]. Breeding, radiographic, clinical, and pathological investigations by DeForest and Basrur [83] supported autosomal dominant transmission of the Manx tailless phenotype with variable expression. Affected cats ranged from animals with reduced or absent caudal vertebrae to individuals with sacral dysgenesis, spina bifida, spinal cord malformations, pelvic-limb locomotor abnormalities, and urinary or fecal incontinence [83]. The external degree of taillessness therefore did not provide a complete measure of internal sacral or neural involvement.

Buckingham et al. [18] subsequently identified four independent frameshift variants in feline *TBXT* in different Manx lineages. Three variants were single-base-pair deletions and one was a small duplication–deletion; each was predicted to truncate the Brachyury protein [18]. Ninety-five percent of the examined Manx cats with shortened-tail phenotypes were heterozygous for one of these variants. Functional reporter testing demonstrated substantially reduced transcriptional activity of mutant Brachyury compared with the wild-type protein [18]. These findings support Brachyury haploinsufficiency as one mechanism underlying the variable Manx short-tail phenotype [18].

The variants identified by Buckingham et al. [18] were associated with the short-tail phenotype, but the study did not establish genotype-based prediction of the extent of sacral or neurological abnormalities in individual cats. Cats with similar degrees of external taillessness may differ in the extent of associated vertebral and neural abnormalities [83]. External taillessness, vertebral morphology, neurological abnormalities, and associated visceral findings represent distinct components of the Manx phenotype [18,83]. The available genetic evidence establishes an association between *TBXT* variants and short-tail morphology but does not define the severity of all associated anatomical or neurological abnormalities in an individual cat [18].

An extreme caudal developmental phenotype in dogs is represented by perosomus elumbis. Amaral et al. [84] described a two-day-old Poodle puppy with absence of the lumbar and sacral vertebrae, hypoplastic and arthrogrypotic pelvic limbs, and absence of the tail. The spinal cord terminated within the thoracic region, the malformed pelvis did not articulate with the remaining vertebral column, and the puppy also had severe intestinal, urinary, and genital abnormalities [84]. This phenotype represented extensive caudal axial agenesis with loss of continuity between the pelvis and the remaining vertebral column rather than an isolated sacral defect [84]. The case illustrates that extensive caudal vertebral agenesis may be accompanied by severe abnormalities outside the vertebral column [84]. The Boxer littermates retained pelvic-limb function despite marked sacral reduction [82], whereas the Poodle puppy described by Amaral et al. [84] lacked the lumbar and sacral vertebrae, had no tail, and exhibited major appendicular and visceral malformations. These phenotypes differ substantially in anatomical extent and functional consequences.

The biological significance of sacral agenesis and sacrocaudal dysgenesis depends substantially on the extent of associated neural involvement. Urinary or fecal incontinence, altered perineal function, and pelvic-limb neurological abnormalities have been documented in affected dogs and Manx cats, while the neurological phenotype varies with the extent and distribution of associated neural abnormalities [62,78,79,80,81,82,83].

Osseous and terminal neural abnormalities represent complementary components of imaging characterization in clinically affected animals. Radiography and computed tomography can characterize vertebral number, residual sacral morphology, sacroiliac relationships, and continuity between the vertebral column and pelvis, whereas magnetic resonance imaging can demonstrate associated abnormalities of the terminal spinal cord, cauda equina, meninges, and related soft tissues [62,78,79,80,81,82,83].

Anatomical characterization of sacral agenesis and sacrocaudal dysgenesis includes the number and morphology of the remaining lumbar, sacral, and caudal vertebrae; the completeness and symmetry of the sacral bodies, wings, and dorsal arches; their relationships with the ilia and caudal spine; and continuity between the vertebral column and pelvis [82,83,84]. Associated neural and systemic abnormalities provide additional components of phenotypic characterization distinct from the osseous morphology. Interpretation also depends on the imaging, pathological, and genetic methods used to define the phenotype. Together, these features help distinguish isolated reduction of the terminal vertebral column from sacrocaudal dysgenesis associated with neural abnormalities and from more extensive multisystem caudal developmental phenotypes.

#### 5.5.2. Caudal Vertebral Number, Congenital Malformations, and Breed-Associated Tail Phenotypes

The caudal region exhibits greater numerical and morphological variability than the more cranial regions of the vertebral column [1,2,32,33,85]. Congenital tail shortening or kinking may result from a reduced number of caudal vertebrae, abnormal formation of individual vertebral bodies, fusion between adjacent vertebrae, altered morphology at the sacrocaudal boundary, or a combination of these configurations [15,16,17,18,19,45,83,85]. External tail length or curvature does not therefore reliably identify the underlying vertebral abnormality [15,17,45,85].

Congenital caudal abnormalities are morphologically distinct from tail shortening or deformation resulting from amputation, trauma, luxation, fracture, or degenerative change [85]. A palpable or externally visible kink may have different anatomical causes, whereas vertebral abnormalities that do not substantially deviate the longitudinal axis of the tail may not be apparent on physical examination [17,45,85]. Clinical history and imaging of the residual tail provide complementary information for distinguishing congenital vertebral morphology from acquired tail shortening or deformation [17,85].

Paninárová et al. [85] prospectively examined the complete caudal vertebral column in two orthogonal radiographic projections in 595 client-owned dogs undergoing hip-dysplasia screening. The study population consisted of client-owned dogs undergoing routine radiographic examination of the hip joints [85]. Normal caudal vertebrae were recorded in 336 dogs, whereas 259 had at least one congenital, morphological, traumatic, or degenerative abnormality, yielding an overall frequency of 43.53%; degenerative changes represented the largest category [85]. Abnormalities classified radiographically as congenital were identified in 66 dogs (11.09%), including sacrocaudal transitional vertebrae in 33 (5.55%), caudal hemivertebrae in 18 (3.03%), and caudal block vertebrae in 15 (2.52%) [85]. No significant association with sex was identified, and none of the breeds represented by more than ten dogs differed significantly from the overall frequency of congenital abnormalities [85]. These frequencies characterize the specific population of dogs examined during radiographic hip screening and therefore reflect the composition of that study cohort [85]. The study also differentiated congenital abnormalities from curved vertebrae and dysplastic vertebral epiphyses, classified as morphological variants, as well as from post-traumatic and degenerative lesions [85]. This radiographic framework provides a morphological classification of caudal abnormalities, although the normal progressive reduction in size and structural complexity of distal caudal vertebrae may complicate classification near the tail apex [1,2,33,85].

Beyond radiographic classification, breed-associated short-tail phenotypes in dogs are genetically heterogeneous [16,17,21]. The canine *TBXT* C189G variant was associated with the natural short-tail phenotype in 17 of 23 breeds examined by Hytönen et al. [21], whereas six naturally short-tailed breeds lacked the variant. This natural bobtail phenotype is genetically and morphologically distinct from the screw tail of Bulldogs, French Bulldogs, and Boston Terriers, which is characterized by shortened and kinked tails with malformed, fused, and reduced numbers of caudal vertebrae [16]. Accordingly, external descriptors such as bobtail, short tail, kinked tail, or screw tail may encompass anatomically and genetically distinct configurations [16,17,18,21,45,83,85].

Mansour et al. [16] identified the frameshift deletion *DVL2* c.2044delC as the variant most strongly associated with the screw-tail phenotype in a whole-genome association analysis of dogs from multiple breeds. The variant was fixed in the Bulldogs and French Bulldogs examined and occurred at high frequency in Boston Terriers [16]. Genotyping and imaging data supported recessive segregation with caudal vertebral malformations, which were fully penetrant among the evaluated homozygous dogs from screw-tail breeds, whereas penetrance of thoracic malformations differed among breeds [16]. Functional experiments demonstrated reduced WNT-dependent phosphorylation of the altered DVL2 protein, supporting altered DVL2-dependent signaling as a contributor to the phenotype [16].

The canine *DVL2* alteration affects a region analogous to that disrupted by *DVL1* and *DVL3* variants in human Robinow syndrome, leading Mansour et al. [16] to propose a Robinow-like syndrome in screw-tail breeds. This comparative interpretation is supported by shared craniofacial, appendicular, and vertebral features but does not imply that every Bulldog-associated vertebral abnormality results directly from *DVL2* [16]. The original whole-genome association compared 10 screw-tail dogs with 84 controls, while the near fixation of the *DVL2* deletion in the defining screw-tail breeds limited within-breed genotype–phenotype comparisons [16].

Niskanen et al. [17] subsequently genotyped 1954 dogs from 15 breeds and found the *DVL2* deletion to be fixed in the 165 Boston Terriers, 297 French Bulldogs, and 211 English Bulldogs examined, while it segregated at lower frequencies in American Staffordshire Terriers, Staffordshire Bull Terriers, Dogues de Bordeaux, Olde English Bulldogges, and American Bulldogs. This variation enabled a more direct assessment of genotype–phenotype relationships in breeds in which the allele was not fixed. The complete tail was evaluated by computed tomography in 19 American Staffordshire Terriers and radiographically in one additional homozygous dog [17]. All four dogs homozygous for the *DVL2* deletion had caudal vertebral malformations, involving one, eight, ten, and fourteen abnormal vertebrae, respectively, with abnormalities including block vertebrae, lateral and dorsolateral hemivertebrae, butterfly vertebrae, ventral wedge-shaped vertebrae, abnormally short vertebrae, and other congenital malformations; one homozygous dog also had sacral vertebral abnormalities [17]. Despite this morphological variation, homozygous dogs retained 20–21 caudal vertebrae, compared with 19–21 in heterozygous and wild-type dogs, indicating that the associated phenotype in this population was expressed predominantly through altered vertebral morphology rather than a consistent marked reduction in caudal vertebral number [17]. Caudal abnormalities were also identified in three of eight heterozygous and two of eight wild-type dogs examined by computed tomography. These findings support *DVL2* as a contributor to caudal vertebral morphology while demonstrating that caudal vertebral malformations also occur independently of *DVL2* homozygosity [17].

The Pug further illustrates the importance of distinguishing external tail carriage from a true screw-tail vertebral phenotype. In the study by Mansour et al. [16], all 29 genotyped Pugs were wild type for the *DVL2* deletion, and their tails were described as full length without the characteristic truncation and caudal malformation pattern identified in Bulldogs, French Bulldogs, and Boston Terriers [16]. The curled tail carriage of the Pug therefore represents a phenotype distinct from the *DVL2*-associated truncated screw-tail morphology characterized in Bulldogs, French Bulldogs, and Boston Terriers [16,17].

A genetically distinct congenital kinked-tail phenotype occurs in Japanese Bobtail cats. Pollard et al. [45] analyzed a pedigree derived from a Japanese Bobtail and obtained results consistent with autosomal dominant inheritance and variable expression of tail length and kink position. Radiographs of the pelvis and tail were obtained from 24 cats, comprising 21 with kinked tails and three with straight tails; in one kinked-tailed cat, the caudal vertebrae could not be counted accurately because of marked tail shortening and superimposition [45]. Kinked-tailed cats generally had fewer caudal vertebrae than straight-tailed cats, with recorded counts ranging from 11 to 21 and a mean of approximately 15.8 [45]. Twelve of the 20 kinked-tailed cats had at least one caudal hemivertebra incorporated into the kink, and caudal block vertebrae were also identified [45]. Histopathological examination in one cat demonstrated that the visible tail scoliosis corresponded to three shortened and malformed caudal hemivertebrae, without histological evidence of degeneration or other acquired abnormalities [45]. The kinks were generally located at a distance from the sacrum, and no associated morbidity or mortality was identified in the relatively young study population [45]. These findings characterize variable anatomical expression within a related pedigree derived from a Japanese Bobtail; the study did not estimate population-level penetrance, and the examined cats were five years of age or younger [45].

The Japanese Bobtail phenotype was not associated with the *TBXT* variants identified in Manx cats [18,45]. The Japanese Bobtail phenotype is therefore genetically and anatomically distinct from Manx sacrocaudal dysgenesis, in which tail reduction may extend to sacral and neural abnormalities associated with locomotor dysfunction or urinary and fecal incontinence [18,83]. No comparable neurological morbidity was identified in the young Japanese Bobtail cohort examined by Pollard et al. [45]. Rather, biological significance depends on the anatomical extent of the malformation, its proximity to the sacrum, and the presence of associated neural or pelvic abnormalities [18,45,83].

Because the spinal cord terminates cranial to the distal caudal vertebrae [1,2,32,33], isolated malformations confined to the distal tail generally have limited potential for direct spinal cord involvement [17,45,85]. Biological or clinical relevance may nevertheless increase when abnormalities involve the proximal tail or sacrocaudal junction or occur together with adjacent neural, meningeal, pelvic, or soft-tissue abnormalities [17,45,83,85]. A visible tail kink may result from different vertebral configurations, and the presence of a congenital caudal malformation alone does not establish the cause of concurrent clinical signs [17,45,85].

Paninárová et al. [85] evaluated the complete residual caudal vertebral column in two orthogonal radiographic projections, permitting differentiation among congenital, morphological, traumatic, and degenerative abnormalities. Computed tomography has been used to characterize three-dimensional caudal and sacral vertebral morphology in dogs with *DVL2*-associated and other tail phenotypes [16,17]. The normal progressive reduction in vertebral size and structural complexity toward the tail apex can limit morphological classification of individual distal vertebrae [1,2,33,85].

Caudal vertebral phenotypes can be characterized by the total number of identifiable vertebrae, vertebral levels involved, direction and degree of axial deviation, morphology of vertebral body formation defects, completeness and symmetry of congenital fusion, configuration of the sacrocaudal junction, and evidence of fracture, luxation, degeneration, or previous amputation [16,17,18,21,45,83,85]. The method used to define a breed-associated phenotype—external examination, radiography, computed tomography, histopathology, or genetic testing—provides additional context for genotype–phenotype comparisons [16,17,18,45,83,85]. This component-based description distinguishes numerical variation from intrinsic vertebral malformation, congenital from acquired tail deformation, and anatomically or genetically distinct phenotypes that may have a similar external appearance. The major congenital and breed-associated vertebral phenotypes discussed in this chapter, together with their principal anatomical characteristics, species or breed associations, and current level of genetic evidence, are summarized in Table 3.

## 6. Integrative Developmental and Genetic Interpretation

Regional vertebral morphology represents the final anatomical outcome of a sequence of developmental processes that includes axial specification, somitogenesis, sclerotome differentiation, resegmentation, chondrogenesis, and ossification [4,5,6,24,31]. Consequently, a postnatal vertebral phenotype does not constitute a direct readout of a single embryological mechanism. Similar morphological configurations may be compatible with disturbances arising at different developmental stages, whereas alteration of a single developmental pathway may affect several vertebral components or axial regions [16,17,18,21,31,63]. Anatomical classification and developmental interpretation represent distinct levels of analysis unless experimental, genetic, or functional evidence directly links them.

This distinction is particularly important for vertebral body formation defects. Terms such as hemivertebra, butterfly vertebra, wedge vertebra, and shortened vertebral body describe postnatal morphology but do not, by themselves, identify the developmental or molecular event responsible for the abnormal vertebral configuration [3,9,11,55,56,57]. Likewise, clustering of thoracic vertebral malformations within Bulldogs, French Bulldogs, Boston Terriers, and Pugs provides evidence of breed association but does not independently establish a specific causal variant [9,10,11,15,16,17,55,56,57]. Developmental or molecular attribution requires evidence beyond radiographic morphology when the underlying mechanism has not been demonstrated.

### 6.1. Genotype–Phenotype Relationships and Levels of Genetic Evidence

The strength and nature of genetic evidence differ substantially among vertebral phenotypes in dogs and cats [16,17,18,21,63,74,75]. At one end of the spectrum are phenotypes for which specific sequence variants have been linked directly to recognizable axial abnormalities. An ancestral variant in canine *TBXT* is associated with natural bobtail morphology in multiple dog breeds, although the same variant is absent from several other breeds with naturally shortened tails [21]. In Manx cats, independent frameshift variants in feline *TBXT* are strongly associated with the characteristic short-tail phenotype and reduced Brachyury function [18]. These studies establish distinct *TBXT*-associated short-tail phenotypes in dogs and Manx cats while also demonstrating that superficially similar short-tail morphologies may have different genetic bases [18,21,45].

A second well-characterized example is the canine *DVL2* c.2044delC frameshift variant. Mansour et al. [16] demonstrated a strong association between homozygosity for this variant and the characteristic screw-tail phenotype of Bulldogs, French Bulldogs, and Boston Terriers. Functional experiments additionally demonstrated reduced WNT-dependent phosphorylation of the altered DVL2 protein, supporting altered DVL2-dependent signaling [16]. Subsequent investigation in American Staffordshire Terriers identified caudal vertebral malformations in all four dogs homozygous for the *DVL2* deletion, while abnormalities were also present in three of eight heterozygous and two of eight wild-type dogs examined by computed tomography [17]. The available evidence therefore supports *DVL2* as an important contributor to the screw-tail and caudal vertebral phenotype but does not justify attribution of every vertebral malformation in these or related breeds to this single variant [16,17].

The genotype–phenotype relationship between *DVL2* and thoracic vertebral body formation defects is less well resolved than that observed for the caudal phenotype [16,17]. Thoracic abnormalities are frequent in several screw-tailed breeds, but their occurrence, anatomical distribution, and morphological subtypes vary substantially among breeds and individuals [16,17,55,56,57]. The presence or high frequency of the *DVL2* deletion in a breed with frequent thoracic vertebral malformations does not demonstrate that the variant directly causes each thoracic hemivertebra or vertebral body formation defect. Current genotype–phenotype evidence is stronger for the caudal vertebral phenotype, whereas the genetic architecture of many thoracic formation defects remains incompletely resolved [16,17].

A more phenotype-specific molecular association has been demonstrated for spinal dysraphism in Weimaraners. Safra et al. [63] performed genome-wide association mapping using four affected and 96 unaffected dogs and identified an approximately 1.5-Mb associated region on canine chromosome 8. Subsequent sequencing identified a frameshift variant, *NKX2-8* A150fs, predicted to truncate the encoded homeobox protein [63]. The variant segregated with the mapped phenotype in the investigated family material, supporting its involvement in this defined breed-associated form of spinal dysraphism. However, the small number of mapped affected dogs and the identification of an additional clinically suspected Weimaraner that did not carry the variant limit generalization beyond the genetically characterized phenotype [63]. The available evidence is therefore specific to the genetically characterized Weimaraner phenotype and does not establish *NKX2-8* A150fs as a determinant of anatomically distinct forms of spina bifida or spinal dysraphism in other breeds [63].

Other vertebral phenotypes show quantitative evidence of inherited susceptibility without identification of a specific causal variant. Lumbosacral transitional vertebrae provide a clear example. Gluding et al. [74] estimated a heritability of h^2^ = 0.27 (standard error = 0.02) for their comprehensive normal-versus-LTV trait definition in German Shepherd Dogs, whereas estimates for the individual LTV categories were lower; positive additive genetic correlations were identified among several LTV categories. Berg et al. [75], using different populations and methodology, obtained breed-specific heritability estimates ranging from 0.056 to 0.314 across nine breeds, including a substantially lower estimate in Norwegian German Shepherd Dogs than that reported in the earlier German Shepherd population. These studies demonstrate substantial variation in heritability estimates across populations, phenotype definitions, and analytical approaches [74,75]. They support an inherited contribution to LTV susceptibility but do not identify a specific causal gene.

Genetic mapping by Brander et al. [70] provides evidence of a complex, multilocus genetic architecture for Pug-associated thoracolumbar myelopathy. Brander et al. [70] analyzed 51 affected and 38 control Pugs using a Bayesian complex-trait mapping approach and identified 19 associated loci containing 67 genes. Candidate genes within these regions were linked to biological functions involving bone and cartilage homeostasis, fibrotic responses, and inflammation [70]. The case definition encompassed the broader clinical thoracolumbar myelopathy phenotype; consequently, the associated loci were mapped to this composite disorder rather than to a specific vertebral, meningeal, or spinal cord lesion [70]. The findings therefore support a multilocus genetic contribution to Pug-associated thoracolumbar myelopathy, while the genetic determinants of individual constituent lesions, including caudal articular process dysplasia and meningeal fibrosis, remain unresolved [69,70].

Familial clustering represents a distinct level of genetic evidence when no causative variant has been identified. The two Boxer littermates with partial sacral agenesis described by Dell’Apa et al. [82] had closely similar congenital abnormalities, supporting the possibility of a shared genetic susceptibility. Whole-genome sequencing of one affected dog, compared with 924 control genomes, did not identify a strong plausible candidate variant under the analytical strategy employed [82]. The occurrence in two littermates remains compatible with an inherited contribution, but neither the mode of inheritance nor the molecular basis was established [82].

Breed predisposition represents a distinct level of evidence from demonstrated heritability or a defined molecular mechanism. High frequencies of vertebral body malformations in several brachycephalic breeds document breed association [9,10,11,15,55,56,57]. The genetic structure of domestic dog breeds and the effects of artificial selection may contribute to the concentration of breed-associated morphological traits [12,13,14], while the molecular basis of these phenotypes may remain unresolved. In the absence of a demonstrated genotype–phenotype relationship, these abnormalities remain breed-associated phenotypes rather than genetically defined disorders.

The same principle applies when developmental candidate genes are inferred from comparative biology. Genes involved in the segmentation clock and related processes of somite boundary formation and axial patterning, including *DLL3*, *MESP2*, *LFNG*, *HES7*, *TBX6*, and *RIPPLY2*, are established determinants of vertebral segmentation disorders in humans and provide biologically plausible candidate mechanisms for analogous developmental disturbances [31]. Their involvement in human disease or experimental models does not demonstrate causation in dogs or cats. Comparative developmental biology therefore provides a framework for generating and prioritizing hypotheses that require subsequent species-specific genetic and, where possible, functional testing.

Genetic evidence for vertebral anomalies can be organized into several levels: species-specific sequence variants supported by genotype–phenotype relationships, with functional evidence where available; quantitative evidence of inherited susceptibility without an identified causal variant; candidate loci identified through association analyses; familial or breed clustering without molecular confirmation; and comparative candidate mechanisms derived from other species. These categories represent non-equivalent levels of genetic evidence. Among the strongest current species-specific molecular associations are *TBXT*-related short-tail phenotypes [18,21], *DVL2*-associated screw-tail and caudal vertebral malformations [16,17], and *NKX2-8* A150fs associated with the genetically characterized Weimaraner spinal dysraphism phenotype [63]. By contrast, the genetic architecture of many thoracic vertebral body formation defects, lumbosacral transitional configurations, Pug-associated thoracolumbar myelopathy, and sacral developmental abnormalities remains incompletely defined [55,56,57,70,74,75,82].

Vertebral morphology, genetic status, and biological significance represent distinct but complementary levels of phenotypic interpretation. Anatomical characterization defines the structural abnormality, genetic evidence establishes the degree of support for inherited susceptibility or a specific molecular determinant, and neurological or biomechanical findings describe the functional context associated with the vertebral phenotype. Considering these levels separately helps distinguish breed association from genetic causation and candidate loci from established molecular mechanisms, while providing a more rigorous framework for future genomic studies based on consistently defined vertebral phenotypes.

### 6.2. Developmental Convergence and Phenotypic Heterogeneity

Congenital vertebral malformations can be classified morphologically as formation defects, segmentation defects, or combined formation–segmentation abnormalities, while veterinary classification systems further distinguish individual configurations according to the morphology and extent of vertebral involvement [3,9,31]. These categories provide a framework for standardized phenotyping but do not establish etiological homogeneity, because morphologically defined vertebral abnormalities may occur within heterogeneous developmental and genetic contexts [31].

Vertebral development encompasses somite formation and boundary specification, sclerotome differentiation and resegmentation, chondrogenesis, and ossification [4,5,6,24,31]. Postnatal morphology does not by itself establish which of these processes was primarily affected. Accordingly, terms such as hemivertebra, wedge vertebra, butterfly vertebra, and block vertebra function primarily as morphological descriptors unless developmental or molecular evidence supports attribution to a more specific mechanism [3,9,31]. This distinction is particularly important when terminology derived from embryological models is applied retrospectively to naturally occurring vertebral abnormalities in dogs and cats.

Phenotypic heterogeneity is also evident within veterinary populations. Brachycephalic dog breeds may exhibit several anatomically distinct vertebral abnormalities, and thoracic vertebral body formation defects themselves comprise multiple radiographically distinguishable configurations [9,10,11,55,56,57]. This diversity demonstrates that congenital vertebral malformations occurring within the same breed may represent anatomically distinct phenotypes. Precise anatomical phenotyping provides a more specific basis for genetic investigation because broad phenotype definitions may combine morphologically distinct vertebral abnormalities.

Within this framework, developmental convergence represents an interpretive concept rather than a demonstrated mechanism for individual canine or feline phenotypes. Similar postnatal morphologies may be compatible with disturbances arising at different stages or components of vertebral development, whereas perturbation of related developmental processes may ultimately produce different anatomical outcomes [4,5,6,24,31]. Consequently, standardized anatomical phenotyping provides the basis for developmentally plausible hypotheses that can subsequently be evaluated through species-specific genetic or functional evidence. This approach preserves the distinction between observed morphology, biologically informed interpretation, and experimentally demonstrated causation.

### 6.3. Breed Association, Genetic Background, and the Effects of Selection

Breed association constitutes a distinct level of evidence for congenital vertebral phenotypes and does not by itself demonstrate a specific mode of inheritance or causal genetic variant. In screw-tailed brachycephalic dogs, both the frequency and morphological spectrum of vertebral malformations differ among breeds. Thoracic vertebral malformations were reported more frequently in neurologically normal French Bulldogs and English Bulldogs than in Pugs, whereas the distribution of individual morphological categories also differed: vertebral body formation defects classified as hemivertebrae were particularly frequent in French Bulldogs, while transitional vertebrae and spina bifida were comparatively more frequent in Pugs [10]. Such breed-specific patterns demonstrate population-level clustering of vertebral phenotypes but do not, by themselves, identify the developmental or genetic mechanisms responsible for these differences.

A stronger level of genetic inference becomes possible when a breed-associated phenotype can be linked directly to a sequence variant. The frameshift deletion *DVL2* c.2044delC provides the clearest canine example relevant to screw-tail morphology. Mansour et al. [16] identified this variant as the most strongly associated genomic variant with the screw-tail phenotype. It was fixed in the Bulldogs and French Bulldogs examined and occurred at high allele frequency in Boston Terriers [16]. Genotype and imaging data supported recessive segregation with caudal vertebral malformations, whereas expression of thoracic vertebral abnormalities varied among the evaluated screw-tail breeds [16]. These distinctions are important because they indicate a stronger genotype–phenotype relationship for the caudal component than for individual thoracic vertebral malformations.

Subsequent investigation across additional breeds further clarified this relationship. Niskanen et al. [17] found the *DVL2* deletion to be fixed in the sampled Boston Terrier, French Bulldog, and English Bulldog populations and demonstrated an association between homozygosity and multiple caudal vertebral malformations in American Staffordshire Terriers [17]. Caudal abnormalities were nevertheless also identified in some heterozygous and wild-type dogs. Together, these findings support *DVL2* as an important contributor to a defined breed-associated axial phenotype rather than as a universal explanation for congenital vertebral malformations or other canine caudal phenotypes [16,17]. The variability of thoracic expression among breeds carrying the *DVL2* deletion is compatible with the influence of genetic background or additional modifying factors, but the relevant modifiers have not been specifically identified [16,17]. No specific modifier genes have yet been demonstrated to account for the breed-dependent differences in thoracic phenotypic expression [16,17].

The converse situation is illustrated by naturally short-tailed dogs, in which similar external phenotypes can have different genetic bases among breeds. The ancestral C189G variant in *TBXT* showed complete correlation with the natural short-tail phenotype in the 17 breeds in which it was detected, whereas naturally short-tailed dogs from six other breeds lacked both this substitution and other coding-region variants in *TBXT* [21]. The canine natural bobtail phenotype is therefore genetically heterogeneous across breeds.

Allelic heterogeneity is also evident in cats, where multiple independent frameshift variants in *TBXT* have been associated with the Manx short-tail phenotype [18]. The Japanese Bobtail phenotype provides a further contrast because the Manx-associated *TBXT* variants were not identified in the pedigree investigated by Pollard et al. [45]. Together, these examples demonstrate that neither a shared external phenotype nor morphological similarity across breeds or species is sufficient to infer a single underlying genetic mechanism.

Artificial selection and breed formation provide additional population-genetic context because breed-defining conformational traits and congenital skeletal phenotypes may occur within the same genetically structured populations [12,13,14]. The *DVL2* deletion is fixed or present at very high frequency in the principal screw-tail breeds and is strongly associated with the screw-tail and caudal vertebral phenotype [16,17]. The available evidence supports an association between the variant and this broader conformational phenotype, while the historical relationship between selection for external morphology and the accompanying vertebral abnormalities remains unresolved. Allele frequency and genotype–phenotype association describe distinct aspects of the genetic architecture of breed-associated traits.

Modern dog breeds are genetically structured populations shaped by breed formation, restricted gene flow, and selection for morphological and other breed-defining traits [12,13,14]. When a variant approaches fixation within a breed, genotype–phenotype relationships may become more difficult to resolve because genetic contrast among individuals is reduced. This situation is relevant to *DVL2* in the principal screw-tail breeds, in which the deletion is fixed or nearly fixed while thoracic vertebral malformations show incomplete and breed-variable penetrance [16,17]. These observations demonstrate that *DVL2* genotype alone does not account uniformly for thoracic vertebral expression across breeds [16,17].

Precise anatomical phenotyping provides the necessary phenotypic resolution for interpreting genetic findings in breed-structured populations. Distinguishing breed-level predisposition from demonstrated genotype–phenotype relationships helps define the biological scope of validated genetic associations and supports more consistent comparison among breeds and vertebral phenotypes [10,16,17,18,21]. External conformational similarity and associated internal skeletal phenotypes do not, by themselves, establish an identical genetic basis or selection history.

## 7. Anatomical, Genetic, and Breeding Implications

The biological significance of congenital and breed-associated vertebral anomalies reflects the anatomical phenotype, its developmental context, and the level of supporting genetic evidence. Structural abnormalities may occur as isolated or incidental findings, form part of a broader breed-associated morphological pattern, or coexist with alterations of vertebral alignment and spinal architecture [3,10,11]. The anatomical variant or malformation, its associated functional consequences, and the strength of evidence for an inherited or specific molecular determinant represent distinct levels of phenotypic interpretation [10,16,17,18,21].

### 7.1. Anatomical Phenotype and Biological Significance

The presence of a congenital vertebral malformation does not, by itself, imply clinically significant spinal disease. This distinction is particularly evident in brachycephalic dogs, in which thoracic vertebral malformations are frequently identified in neurologically normal individuals [10,11]. Consequently, frequencies derived from diagnostic imaging primarily describe the occurrence of structural phenotypes within the populations examined and do not necessarily represent the frequency of clinically significant spinal disease.

From an anatomical perspective, the biological significance of a vertebral abnormality depends not only on its morphological classification but also on its regional location, extent, symmetry, and relationship to the overall architecture of the vertebral column [3,9,10,11]. A localized abnormality may preserve vertebral alignment and canal dimensions, whereas more extensive or asymmetrical configurations may coexist with angular deformation, altered relationships between adjacent vertebral segments, or narrowing of the vertebral canal [3,9,10,11]. Morphological type, anatomical region, extent, symmetry, and associated spinal architecture therefore provide information beyond the presence or number of malformed vertebrae alone.

The same distinction is important for developmental and genetic investigation. Clinical status provides information complementary to anatomically resolved phenotyping. Animals grouped under a broad category such as “vertebral malformation” may have morphologically distinct abnormalities that are not necessarily developmentally or genetically equivalent [9,10,11,31]. Combining morphologically distinct abnormalities within a single phenotype category may reduce phenotypic resolution in developmental or genetic analyses.

An anatomically resolved phenotype definition includes the type of vertebral abnormality, anatomical level, regional distribution, symmetry, and, where relevant, associated alteration of vertebral alignment. Neural, meningeal, intervertebral disc, and other associated pathological abnormalities represent components distinct from the congenital osseous phenotype. This component-based description separates congenital osseous morphology from associated neural, meningeal, intervertebral, or other pathological findings and provides greater phenotypic resolution for genotype–phenotype analysis.

Anatomical precision is particularly important when externally similar phenotypes have different underlying vertebral configurations. Shortened, kinked, bobbed, or screw tails may reflect variation in caudal vertebral number, vertebral body formation defects, congenital fusion, altered sacrocaudal morphology, or combinations of these abnormalities [16,17,18,21,45,83,85]. Similarly, transitional morphology at regional boundaries is anatomically distinct from true numerical variation and from intrinsic vertebral formation or segmentation abnormalities. Phenotypic classification based predominantly on external appearance or broad diagnostic labels may therefore be insufficient for developmental or genetic interpretation.

Biological significance can be described at several related but distinct levels: the anatomical vertebral phenotype, its structural relationships within the vertebral column, associated neural or soft-tissue abnormalities, and the functional findings documented in the individual animal or study population. These distinctions separate incidental congenital morphology from clinically significant disease while retaining the anatomical relevance of abnormalities associated with angular deformation, neural compromise, or other structural changes.

### 7.2. Implications for Genetic Investigation and Breeding

Accurate anatomical phenotyping is fundamental to the genetic investigation of congenital vertebral anomalies. Morphologically distinct abnormalities may occur within the same breed and may reflect disturbances affecting different components or stages of vertebral development [9,10,11,31]. Phenotypic resolution with respect to vertebral morphology, anatomical region, distribution, symmetry, and associated structural changes therefore provides a more informative basis for genetic analysis than grouping heterogeneous vertebral abnormalities within a single category.

The biological significance of a genetic variant is likewise closely linked to the phenotype and population in which its association has been demonstrated. The *DVL2* c.2044delC frameshift variant provides a clear example: homozygosity is strongly associated with the screw-tail morphological complex and caudal vertebral malformations in relevant canine populations, whereas thoracic vertebral abnormalities show variable expression among breeds and caudal abnormalities may also occur outside the homozygous genotype [16,17]. The established *DVL2* association is specific to the axial phenotypes and canine populations in which the genotype–phenotype relationship has been demonstrated [16,17].

A comparable principle is illustrated by *TBXT*. The canine *TBXT* C189G variant is strongly associated with natural bobtail morphology in multiple breeds but is absent from several other breeds with naturally shortened tails [21]. In cats, several independent frameshift variants in *TBXT* are associated with the Manx short-tail phenotype [18], whereas the kinked-tail phenotype investigated in Japanese Bobtail cats was not associated with these Manx variants [45]. Thus, phenotypic similarity does not necessarily imply allelic identity or even involvement of the same gene.

These observations have direct implications for the interpretation of genetic information in breeding populations. Interpretation of a validated genotype–phenotype association includes the anatomical phenotype, inheritance pattern, and population in which the association was characterized. Incomplete penetrance, variable expression, genetic heterogeneity, and near fixation of an associated allele can limit the correspondence between genotype at a single locus and the complete vertebral phenotype [16,17,18,21]. Breeding-oriented interpretation therefore encompasses several distinct levels of evidence, including specific variants with demonstrated genotype–phenotype relationships, quantitative evidence of inherited susceptibility without an identified causal variant, breed-level or familial clustering, and anatomically defined abnormalities whose genetic basis remains unresolved.

This distinction is particularly relevant to lumbosacral transitional vertebrae and Pug-associated thoracolumbar myelopathy. Pedigree-based studies support an inherited component and measurable heritability for lumbosacral transitional vertebrae in several canine populations [74,75], whereas the underlying molecular determinants remain unresolved. Phenotypic and genetic studies of Pug-associated thoracolumbar myelopathy support a heterogeneous clinical disorder with complex genetic susceptibility rather than a single molecular determinant for caudal articular process dysplasia or any individual spinal cord lesion [69,70]. In such settings, genetic evidence and standardized anatomical classification provide complementary levels of phenotypic interpretation.

Breed association represents another distinct form of evidence. Increased frequency of a vertebral abnormality within a breed may reflect population structure, correlated conformational traits, inherited determinants, or combinations of factors that remain incompletely resolved [12,13,14,15,16,17]. Anatomical phenotype, clinical significance, breed context, and molecular genotype provide complementary information for breeding-oriented and genetic research. Within this framework, established genetic associations retain their value within the phenotypes and populations in which they have been demonstrated, while unresolved vertebral abnormalities remain open to further genetic and developmental investigation.

## 8. Knowledge Gaps and Research Priorities

Taken together, the current evidence base is limited by heterogeneous phenotype definitions and classification schemes, the predominance of retrospective, breed-specific, referral, or otherwise selected populations, uneven sample sizes, limited species-specific molecular evidence for many abnormalities, particularly in cats, and incomplete genotype–phenotype relationships even for several breed-associated canine conditions. These limitations restrict direct comparison among studies, complicate population-level estimates of phenotype frequency and risk, and can blur the distinction among breed enrichment, heritable susceptibility, and validated molecular causation. They also explain why standardized anatomical phenotyping, prospectively characterized populations, species-specific genomic investigation, and functional validation represent central research priorities.

Anatomical phenotyping therefore requires greater standardization. Differences in anatomical classification and phenotype definition can limit direct comparison among study populations. Existing classification systems provide useful frameworks for distinguishing vertebral formation and segmentation abnormalities, while detailed characterization of morphology, anatomical level, regional distribution, vertebral number, symmetry, and transitional identity provides a more consistent basis for phenotypic comparison [9,11,31]. Reproducible anatomical definitions would allow structurally distinct abnormalities to be evaluated as separate phenotypes rather than combined within broad diagnostic categories.

Study population design represents another important consideration for future work. Several influential veterinary studies have been retrospective or have examined breed-specific or otherwise selected populations, including referral cohorts, neurologically normal animals undergoing imaging for unrelated indications, and pedigreed animals selected for particular morphological traits [9,10,45]. These study designs have contributed substantially to the characterization of phenotypic diversity and to the generation of developmental and genetic hypotheses. Population-level characterization would benefit from larger, prospectively defined and appropriately breed-stratified cohorts, which could provide more precise estimates of phenotype frequency and allow anatomically distinct subtypes to be evaluated individually.

Genetic investigation would similarly benefit from closer alignment with anatomically resolved phenotypes. The associations involving *DVL2* and *TBXT* demonstrate that specific variants can be associated with well-defined components of vertebral or tail morphology, while apparently similar phenotypes may remain genetically heterogeneous across breeds and populations [16,17,18,21]. Future genotype–phenotype studies combining precise anatomical classification with well-characterized population structure could clarify the genetic architecture of unresolved phenotypes and define more precisely the phenotypic range associated with established variants. Such an approach would also facilitate comparison among morphologically related abnormalities that may arise through distinct genetic mechanisms.

Variation in phenotypic expression among animals carrying the same associated variant provides an additional opportunity to investigate the contribution of genetic background and modifying loci [16,17]. Genetic background and modifying loci therefore remain testable hypotheses for variable expression rather than demonstrated determinants [16,17]. Populations containing sufficient numbers of genotyped and anatomically phenotyped animals, particularly where the associated allele remains polymorphic, provide greater genetic contrast for investigating variable expression. Cross-breed comparisons may provide complementary information when anatomical definitions and imaging protocols permit consistent phenotypic comparison.

A further research priority concerns the relationship between statistical genetic association and developmental mechanism. Functional investigation of the *DVL2* frameshift variant and mutant *TBXT* alleles illustrates how molecular assays can strengthen genotype–phenotype interpretation by linking sequence variation with altered protein function [16,18]. Comparable functional approaches may be particularly valuable for candidate genes emerging from comparative developmental biology. Genes involved in somitogenesis, somite boundary formation, rostrocaudal polarity, resegmentation, and vertebral regional identity provide biologically plausible candidates, and species-specific genetic and functional investigation could clarify their relevance to individual canine and feline phenotypes [31]. Integration of developmental biology with veterinary genomics therefore offers a framework for progressing from biological plausibility toward experimentally supported mechanisms.

Phenotypic resolution can also extend beyond vertebral body morphology. Transitional vertebrae, dorsal arch defects, articular process dysplasia, congenital fusion, sacral abnormalities, and caudal vertebral phenotypes may represent biologically distinct traits even when they occur within the same animal or breed. Individual-level integration of anatomical and genetic data could help determine whether such abnormalities represent components of a shared developmental-genetic phenotype or distinct traits that cluster within genetically structured populations. This distinction may be particularly informative in breeds in which several congenital axial abnormalities occur concurrently.

Longitudinal characterization represents another important opportunity. Many studies cited in this review characterize vertebral morphology within retrospective or cross-sectional imaging datasets, whereas longitudinal structural and functional evolution has been evaluated less frequently [9,10,37,45,55,65,76]. Prospective longitudinal studies could clarify which configurations remain clinically silent and which become associated with changes in vertebral alignment, adjacent intervertebral structures, meninges, or neural tissues. Such data could help distinguish congenital osseous morphology from later-acquired structural or pathological changes and clarify the temporal relationship between anatomical configuration and functional outcome.

Within the feline literature reviewed here, species-specific molecular evidence is concentrated primarily in the Manx short-tail phenotype. Independent frameshift variants in feline *TBXT* provide the principal established molecular association, whereas the causal genetic basis of the Japanese Bobtail kinked-tail phenotype and the other feline vertebral abnormalities discussed in this review remains unresolved [18,45]. Anatomical studies have documented additional feline phenotypes, including transitional vertebrae, numerical variation, block vertebrae, vertebral body formation defects, and spinal dysraphism [37,54,73,76,80]. Integration of anatomically characterized feline phenotypes with genomic investigation could determine whether genetic determinants identified for defined canine phenotypes have feline counterparts or whether distinct species-specific mechanisms are involved.

Future studies integrating standardized anatomical phenotyping, developmental interpretation, appropriately designed population analyses, genetic association, and functional validation could address several of the unresolved questions identified in this review. Such integration provides a coherent framework for distinguishing anatomical description, developmental hypothesis, and demonstrated molecular causation, while advancing understanding of the biological basis of congenital vertebral diversity in dogs and cats.

## 9. Conclusions

Congenital and breed-associated vertebral abnormalities in dogs and cats represent a heterogeneous group of phenotypes involving vertebral formation, segmentation, regional identity, and number. Similar external or radiographic appearances may arise through different developmental and genetic mechanisms; consequently, precise anatomical phenotyping is essential for meaningful comparison among studies and for genotype–phenotype analysis. Morphological description should therefore remain distinct from inference about embryological mechanism or molecular causation.

Defined molecular associations, particularly those involving *DVL2* and *TBXT*, have clarified the genetic basis of specific axial phenotypes, but the genetic determinants of many canine and especially feline vertebral abnormalities remain unresolved. Breed association, heritability, and demonstrated molecular causation represent distinct levels of evidence. Progress will depend on standardized anatomical definitions, larger and appropriately structured populations, species-specific genomic studies, and functional validation of candidate mechanisms, providing a more rigorous basis for biological interpretation and breeding-oriented application.

## Figures and Tables

**Table 1:** Developmental processes, morphological outcomes, and genetic evidence relevant to vertebral malformations in dogs and cats.

Developmental Process	Principal Biological and Molecular Context	Representative Vertebral Outcomes	Evidence and Interpretation in Dogs and Cats	References
Somite formation and boundary specification	Periodic segmentation of the presomitic mesoderm through the segmentation clock and wavefront mechanism; coordinated Notch, WNT/β-catenin, and fibroblast growth factor signaling	Altered segment number, abnormal segment boundaries, bilateral asymmetry, and complex vertebral segmentation defects	Mechanistic understanding is derived primarily from experimental and comparative developmental evidence, while species-specific genetic causation remains unresolved for many common canine and feline vertebral phenotypes	[11,25,26]
Sclerotome differentiation and vertebral resegmentation	Migration and differentiation of sclerotomal cells around the notochord and neural tube; coordinated formation of vertebral bodies, neural arches, ribs, and intervertebral boundaries	Incomplete vertebral separation, congenital fusion, block vertebrae, asymmetrical fusion, hypoplastic or absent vertebral components	Supported by developmental anatomy and veterinary morphology. Similar lesions may arise through different disturbances of sclerotomal contribution or resegmentation	[4,5,6,24]
Vertebral body formation and chondrogenesis	Formation of the centrum and cartilaginous vertebral template under the influence of Sonic hedgehog, bone morphogenetic protein, WNT, and related chondrogenic pathways	Hypoplastic, wedge-shaped, butterfly-shaped, or asymmetrical vertebral bodies; lesions traditionally grouped as “hemivertebrae”	Anatomical and morphological evidence exists in dogs and cats, whereas the genetic basis remains unresolved for many vertebral body malformations. Similar morphology may reflect more than one embryological mechanism.	[3,4,5,6,24]
Neural arch formation and dorsal closure	Formation and fusion of the dorsal vertebral components in close spatial relationship with the neural tube and meninges	Neural arch aplasia or hypoplasia, incomplete dorsal closure, spina bifida, and spinal dysraphism	Documented in dogs and cats, sometimes together with spinal cord or meningeal abnormalities. Molecular determinants are generally unresolved in veterinary species	[3,4,5,6,24]
Regional axial identity	Craniocaudal specification of vertebral identity through coordinated *HOX* gene expression and regional patterning	Cervical ribs, thoracization of C7, thoracolumbar or lumbosacral transitional vertebrae, and mixed regional morphology	Mechanistic evidence is mainly comparative, but homeotic transformation of C7 and associated cervical ribs have been documented in dogs, particularly Pugs	[7,8,27,28,29,30]
Comparative segmentation-gene disorders	Pathogenic variation in segmentation-clock genes and related regulators, including *DLL3*, *MESP2*, *LFNG*, *HES7*, *TBX6*, and *RIPPLY2*	Spondylocostal dysostosis, congenital scoliosis, vertebral fusion, and complex segmentation abnormalities in humans	These genes identify biologically relevant developmental pathways but should be regarded as comparative or candidate determinants in dogs and cats unless species-specific genetic evidence is demonstrated	[31]
WNT-associated breed phenotype: *DVL2*	Frameshift variation in the *DVL2* gene, which encodes the Dishevelled 2 protein, a component of WNT signaling involved in axial morphogenesis	Screw-tail morphology and a broader Robinow-like constellation of axial skeletal characteristics	Validated canine association in Bulldogs, French Bulldogs, and Boston Terriers. Its association is specific to defined breed-associated phenotypes and does not account for all vertebral or shortened-tail phenotypes	[16,17]
Posterior axial development: *TBXT*	*TBXT* encodes a T-box transcription factor involved in notochord formation, axial mesoderm development, and posterior body-axis formation	Manx, tailless, and short-tail phenotypes in cats; natural bobtail phenotypes in dogs; variable sacrocaudal involvement	Validated species-specific associations. In cats, phenotypic expression and associated sacrocaudal or neural abnormalities are variable. In dogs, the same variant is not present in all naturally short-tailed breeds	[18,19,20,21]

**Table 2:** Regional anatomy of the vertebral column in dogs and cats as a framework for developmental and pathological interpretation.

Vertebral Region	Normal Pattern in Dogs and Cats	Principal Anatomical Characteristics	Developmental and Pathological Relevance	References
Cervical	Seven vertebrae, C1–C7	Highly specialized atlas and axis; C3–C6 conform more closely to the general vertebral pattern; C7 forms the cervicothoracic boundary	Strong developmental constraint on cervical number; the atlas and axis are vulnerable to malformations of specialized components and craniocervical articulation; altered C7 regional identity is morphologically associated with cervical ribs or cervicothoracic transitional morphology	[1,2,3,28,29,30,32,33]
Thoracic	Thirteen vertebrae, T1–T13	Articulation with the ribs through costal and transverse costal foveae; long spinous processes; anticlinal vertebra usually T11; progressive transition toward lumbar morphology caudally	Integration with the rib cage means that asymmetric vertebral body formation may affect spinal curvature, rib alignment, and thoracic symmetry; frequent location of vertebral body malformations in brachycephalic screw-tailed dogs	[1,2,3,9,10,11,15,16,17,32,33,34]
Lumbar	Seven vertebrae, L1–L7	Large vertebral bodies; prominent transverse, articular, mammillary, and accessory processes; mobile load-bearing region between the thoracic spine and sacrum	Transitional morphology at the thoracolumbar and lumbosacral boundaries is compatible with altered regional identity and may involve ribs, transverse processes, or variable incorporation into the sacrum	[1,2,3,7,8,27,32,33,35,36,37]
Sacral	Three fused vertebrae, S1–S3	Short, compact sacrum; broad cranial base and narrower caudal apex; paired wings; sacral canal and foramina; articulation with the ilia through the sacroiliac joints	Developmental and biomechanical interface between the lumbar column, pelvis, and caudal series; incomplete lumbar–sacral specification may produce lumbosacral transitional vertebrae; posterior axial disturbances may extend across the sacrocaudal boundary	[1,2,3,7,8,18,19,27,32,33,35,36,37]
Caudal	Variable number, generally approximately 20 vertebrae in both species	Progressive proximodistal reduction of vertebral arches and processes; relatively mobile intervertebral articulations; haemal processes or chevrons may occur proximally	Combines normal numerical variability with susceptibility to posterior axial malformations; genetically associated phenotypes include Manx short-tail or taillessness, canine natural bobtail, and screw-tail morphology	[1,2,3,16,17,18,19,20,21,32,33]

C, cervical vertebrae; T, thoracic vertebrae; L, lumbar vertebrae; S, sacral vertebrae; Cd, caudal vertebrae.

**Table 3:** Major congenital and breed-associated vertebral phenotypes in dogs and cats: anatomical distribution, morphological characteristics, and genetic evidence.

Anatomical Region	Major Phenotype	Principal Morphological Characteristics	Main Species/Breed Associations	Genetic Evidence	References
Craniocervical junction	Atlas-axis and dens abnormalities	Dens aplasia/hypoplasia or malformation; incomplete atlas ossification; occipitoatlantal abnormalities; instability	Mainly small- and toy-breed dogs; sporadic feline cases	No general causal variant established	[3,38,39,40,41,42,43,44]
Cervicothoracic junction	Cervicothoracic transitional vertebra/cervical ribs	Thoracic-like C7 with unilateral or bilateral rib-like structures; segmental separation preserved	Particularly frequent in Pugs in imaging cohorts; also reported in cats	Compatible with altered regional identity; no causal variant established	[7,8,27,28,29,30,45]
Cervical/thoracic dorsal elements	Dorsal arch defects, bifid spinous processes, spina bifida, spinal dysraphism	Dorsal arch non-closure or bifid processes, with variable meningeal/neural involvement	T1 dorsal abnormalities frequent in Pugs; sporadic canine and feline dysraphism	*NKX2–8* A150fs is associated with Weimaraner dysraphism; not established for Pug T1 defects or other forms	[10,30,46,47,48,62,63,64]
Cervical region	Congenital block vertebrae	Congenital fusion/loss of segmentation between adjacent vertebrae, with variable arch involvement	Sporadic in dogs; localized and multilevel forms reported	No specific causal variant established	[3,49,50,51]
Thoracic region	Vertebral body formation defects	Vertebral body aplasia/hypoplasia; wedge, butterfly, shortened, or asymmetrical configurations	French and English Bulldogs, Pugs, and other brachycephalic breeds	*DVL2* contributes to the screw-tail complex but does not explain all thoracic defects	[9,10,11,15,16,17,55,56,57,58,59]
Thoracic region	Block vertebrae	Congenital fusion/loss of segmentation of adjacent thoracic vertebrae, with variable arch involvement	Especially French and English Bulldogs; sporadic feline cases	No specific causal variant established	[3,9,10,54,60,61]
Caudal thoracic region	Caudal articular process dysplasia	Hypoplasia or aplasia of caudal articular processes, often bilateral and multilevel	Very frequent in Pugs; also documented in French and English Bulldogs	No gene established for the osseous defect; multilocus susceptibility reported for broader Pug thoracolumbar myelopathy	[11,65,66,67,68,69,70]
Lumbosacral junction	Lumbosacral transitional vertebra	Mixed lumbar-sacral identity; symmetric or asymmetric transverse processes/sacral wings	Dogs, including several screened breeds such as German Shepherd Dogs; also cats	Heritability demonstrated in canine populations; no causal variant established	[36,37,71,72,73,74,75]
Lumbar region	Vertebral body formation defects	Focal vertebral-body hypoplasia/aplasia or wedge/asymmetry, sometimes with angular deformation	Sporadic canine cases; very limited feline evidence	No specific genetic determinant established	[3,9,11,76,77]
Lumbar/lumbosacral region	Spina bifida and spinal dysraphism	Dorsal arch defects with variable meningeal, neural, dermal, or tethered-cord involvement	Bulldogs and sporadic cases in other dogs and cats	No general causal variant established	[46,62,78,79,80]
Sacral and sacrocaudal region	Sacral agenesis and sacrocaudal dysgenesis	Sacral reduction/agenesis and abnormal sacrocaudal continuity, with variable neural or visceral involvement	Two Boxer littermates; established Manx-associated phenotype	Boxer: no strong candidate variant identified; Manx: *TBXT* frameshift variants established	[18,82,83]
Caudal region	Natural bobtail, screw tail, and kinked-tail phenotypes	Reduced caudal vertebral number, malformation, fusion, and/or axial deviation	Bobtail dog breeds; screw-tail Bulldogs/French Bulldogs/Boston Terriers; Japanese Bobtail cats	*TBXT*: natural bobtail in many dog breeds; *DVL2*: screw tail; Japanese Bobtail not explained by Manx *TBXT* variants	[16,17,18,21,45,83,85]

**Note:** Morphologically similar vertebral phenotypes may arise through different developmental processes or genetic mechanisms. “No specific causal variant established” denotes the absence of a validated species-specific molecular determinant for the phenotype considered here and does not exclude genetic susceptibility. Breed association, heritability, candidate-gene evidence, association loci, and genotype–phenotype relationships represent distinct levels of genetic evidence.

## Data Availability

No new data were created or analyzed in this study. Data sharing is not applicable to this article.
